# Remarks on *Mastigodiaptomus* (Calanoida: Diaptomidae) from Mexico using integrative taxonomy, with a key of identification and three new species

**DOI:** 10.7717/peerj.8416

**Published:** 2020-01-29

**Authors:** Martha A. Gutiérrez-Aguirre, Adrián Cervantes-Martínez, Manuel Elías-Gutiérrez, Alfonso Lugo-Vázquez

**Affiliations:** 1 Departamento de Ciencias y Humanidades, Universidad de Quintana Roo, Cozumel, Quintana Roo, Mexico; 2 Departamento de Sistemática y Ecología Acuática, El Colegio de la Frontera Sur, Chetumal, Quintana Roo, Mexico; 3 Unidad de Investigación Interdisciplinaria en Ciencias de la Salud y la Educación, Universidad Nacional Autónoma de México, Los Reyes Iztacala, Tlalnepantla, Estado de México, Mexico

**Keywords:** COI gene, Freshwater, Barcodes, Zooplankton, Copepoda, Neotropical

## Abstract

**Background:**

In Mexico, species of four families of free-living calanoid copepods have been recorded as inhabitants of several freshwater systems. These families are Centropagidae, Temoridae, Pseudodiaptomidae and Diaptomidae. The genera *Leptodiaptomus* and *Mastigodiaptomus* are the most speciose diaptomid genera in Mexico, and they inhabit natural and artificial lakes, ephemeral ponds, springs, and caverns. *Leptodiaptomus* is considered as an endemic Nearctic genus, whereas *Mastigodiaptomus* is a widely distributed Neotropical genus in the southern USA, Mexico, the Caribbean Islands and Central America. Based on new and recent evidence, *Mastigodiaptomus* diversity has been underestimated: six species of the genus were known before 2000. In this work three new *Mastigodiaptomus* species have been described from different regions of Mexico by using integrative taxonomy. We also gave amended diagnosis of *M. nesus* Bowman (1986) and *M. patzcuarensis* s. str. (Kiefer, 1938).

**Methods:**

In this work, the taxonomic status of the species was clarified using modern, integrative method based on the COI gene as a DNA marker, plus micro-structural analysis (based on SEM and ligth microscopy).

**Results:**

Three new species of *Mastigodiaptomus* were described based on genetic and morphological analyses: *M. alexei* sp. n., *M. ha* sp. n. and *M. cihuatlan* sp. n. Also amended description of *M. nesus*, morphological variation of *M. patzcuarensis* s. str., and a comparison of them with all known sequences within the genus are provided. These new findings show that in *Mastigodiaptomus* differences in several cuticular microstructures of several appendages (such as the antennules, the fifth legs, or the urosomites of these copepods) agree with the interspecific genetic divergence >3% observed in sequences of the COI gene, and the integration of this information is a powerful tool in species delineation.

## Introduction

In Mexico, species of four families of free-living calanoid copepods have been recorded as inhabitants of several freshwater systems. These families are Centropagidae, Temoridae, Pseudodiaptomidae and Diaptomidae.

The genera *Leptodiaptomus* and *Mastigodiaptomus* are the most speciose diaptomid genera in Mexico, and they inhabit natural and artificial lakes, ephemeral ponds, springs, and caverns. *Leptodiaptomus* is considered as an endemic Nearctic genus, whereas *Mastigodiaptomus* is a widely distributed Neotropical genus in the southern USA, Mexico, the Caribbean Islands and Central America.

Based on new and recent evidence, *Mastigodiaptomus* diversity has been underestimated: six species of the genus were known before 2000 (Suárez-Morales & Elías-Gutiérrez, 2000). Recently, however, five new *Mastigodiaptomus* species have been described from different regions of Mexico by using integrative taxonomy (Suárez-Morales & Elías-Gutiérrez, 2000; Gutiérrez-Aguirre & Cervantes-Martínez, 2013; Gutiérrez-Aguirre, Cervantes-Martínez & Elías-Gutiérrez, 2014; Mercado-Salas et al., 2018).

Under this assumption, in this paper, three new species of *Mastigodiaptomus* are described based on genetic and morphological analyses. In addition, amended description of *Mastigodiaptomus nesus* Bowman, 1986 and *M. patzcuarensis* (Kiefer, 1938) and a comparison of them with all known sequences within the genus is provided. Finally, a key (based on the morphology) for the identification of the known species of the genus is yielded.

## Materials and Methods

### Morphological analysis

Biological samples were obtained by limnetic plankton trawls using a plankton net with 50 μm mesh, and the material was fixed in 96% ethanol. The surveyed samples were collected along a latitudinal gradient from 17° to 24° N and a longitudinal gradient from 87° to 101° W. A full morphological description of adult males and females of the three new species, as well as an amended description of one more species of *Mastigodiaptomus*, is presented: the morphology of paratype specimens of *M. nesus* was analysed.

Detailed analysis and illustration of morphology were performed with the aid of a camera lucida, light microscopy and the scanning electron microscopy JEOL-SM-6010, located at El Colegio de la Frontera Sur (ECOSUR, Chetumal Unit) according to Suárez-Morales et al. (1996).

Several analysed specimens were dissected with tungsten needles, and their appendages were mounted in glycerine. The un-dissected biological material was preserved in 100% ethanol with a drop of glycerine. The type material and specimens were deposited in the Reference Collection at El Colegio de la Frontera Sur (ECOCH-CH-Z).

The abbreviations for the appendages or anatomical micro-structure of the body of the adult females and males analysed here are based on those used by Huys & Boxshall (1991), Gutiérrez-Aguirre & Cervantes-Martínez (2016) and Mercado-Salas et al. (2018):

A1 = antennule; Enp1–Enp3 = first to third endopodal segment; Exp1–Exp3 = first to third exopodal segment; P1–P5 = legs 1–5; Bsp = basipodite; s = setae; sp = spine; sps = spiniform process; ae = aesthetasc; ms = modified seta.

### DNA marker and extraction, PCR products and sequencing

The COI gene, known as DNA barcode (Hebert et al., 2003), was here used as a marker to delimit the species as in previous studies (Gutiérrez-Aguirre, Cervantes-Martínez & Elías-Gutiérrez, 2014; Mercado-Salas et al., 2018). DNA was extracted using a standard glass fibre method (Ivanova, DeWaard & Hebert, 2006) from dorsal muscles of the cephalothorax or eggs (in egg-carrying females) following Elías-Gutiérrez et al. (2018). In all cases, the vouchers were deposited in the Reference Collection at El Colegio de la Frontera Sur, Unidad Chetumal (Access Numbers ECOCH-CH-Z-10312 to 10328). All sequences were uploaded to the Barcode of Life Database (BOLD, boldsystems.org) and GenBank (https://www.ncbi.nlm.nih.gov/genbank/).

DNA extraction: 2 μl of each DNA extract was added to a PCR mixture consisting of 2 μl of HyClone ultra-pure water (Thermo Fisher Scientific, Waltham, MA, USA), 6.25 μl of 10% D-(+)-trehalose dihydrate (Fluka Analytical, Munich, Germany), 1.25 μl of 10× Platinum Taq buffer (Invitrogen, Carlsbad, CA, USA), 0.625 μl of 50 μM MgCl_2_ (Invitrogen, Carlsbad, CA, USA), 0.0625 μl of 10 μM dNTP (KAPA Biosystems, Wilmington, MA, USA), 0.125 μl each of 10 μM Zplank primers (Prosser, Martínez-Arce & Elías-Gutiérrez, 2013), and 0.06 μl of PlatinumTaq (Invitrogen, Carlsbad, CA, USA). The reactions were cycled at 95 °C for 1 min, followed by 5 cycles of (94 °C for 40 s, 45 °C for 40 s and 72 °C for 1 min), 35 cycles of (94 °C for 40 s, 51 °C for 40 s and 72 °C for 1 min), and a final extension of 72 °C for 5 min.

Products: PCR products were visualised on a 2% agarose gel using an E-Gel 95-well Pre-cast Agarose Electrophoresis System (Invitrogen, Carlsbad, CA, USA), and the detected PCR products were selected for sequencing. Products were labelled with a BigDye© Terminator v. 3.1 Cycle Sequencing Kit (Applied Biosystems, Inc., Foster City, CA, USA) and sequenced bidirectionally on an ABI 3730 capillary sequencer. Sequence data, trace files, collection data, and primer details for all specimens are available within the two project files in the Barcode of Life Data System (http://www.barcodinglife.org) and in GenBank. Bidirectional sequences were assembled and simultaneously aligned in SeqScape 2.1.1 (Applied Biosystems, Foster City, CA, USA).

All sequences were searched against the BOLD (boldsystems.org). Sequences of closely related species were downloaded from the system and included in the analyses. With all sequences the public dataset with the name *Mastigodiaptomus* was created in BOLD database (DOI: 10.5883/DS-MMASTIGO).

With the Alignment Transformation Environment (ALTER, http://www.sing-group.org/ALTER/), representative haplotypes of all the genetic variants of COI within and between species were obtained (Supplemental File 1). The FASTA files obtained were then introduced into PGDSpider Vers. 2.1.1.5 (http://www.cmpg.unibe.ch/software/PGDSpider/) to obtain the nexus file compatible with MrBayes Vers. 3.2.5 and jModelTest 2.1.10. The best fit was HKY+G, and it was used in MrBayes along with 1 M generations to obtain a tree (Supplemental File 2). The general mixed Yule coalescent (GMYC) model was used as a species delimitation method in which the simple threshold approach assumes that there is a threshold time before which all nodes reflect diversification events (interspecific) and after which all nodes reflect coalescent events (intraspecific). The number of species obtained by this approach is thus estimated by this threshold time. The GMYC method was applied in the ‘splits’ R package for the ultrametric COI tree, obtained with BEAST v1.8.3.

### Nomenclatural acts

‘The electronic version of this article in Portable Document Format will represent a published work according to the International Commission on Zoological Nomenclature (ICZN), and hence the new names contained in the electronic version are effectively published under that Code from the electronic edition alone. This published work and the nomenclatural acts it contains have been registered in ZooBank, the online registration system for the ICZN. The ZooBank Life Science Identifiers (LSIDs) can be resolved and the associated information viewed through any standard web browser by appending the LSID to the prefix http://zoobank.org/. The LSID for this publication is: urn:lsid:zoobank.org:pub:FEC66E33-E192-41C6-B3E1-C77C5D6705DE. The online version of this work is archived and available from the following digital repositories: PeerJ, PubMed Central and CLOCKSS’.

## Results

All the specimens analysed here were classified as species of *Mastigodiaptomus* and showed the following armature in the trunk limbs: first leg (P1) with 3-segmented Exp and 2-segmented Enp. Trunk limbs P2–P4 with 3-segmented Exps and Enps. Exp1–Exp3 of P1–P4 with one lateral spine (Table 1). In addition, the analysed specimens showed the morphological features that define *Mastigodiaptomus* according to Kiefer (1932), Wilson & Yeatman (1959) and Dussart & Defaye (1995).

**Table 1:** Ornamentation of prosomal appendages. Armament of swimming legs (P1–P4) in the females and males of the *Mastigodiaptomus* species described here. Roman numerals indicate spines, and arabic numbers are setae.

	Coxa	Basis	Exp	Enp
P1 P2 P3 P4	0-1 0-1 0-1 0-1	0-0 0-0 0-0 1-0	I-1; 0-1; I-3-2 I-1: I-1; I-3-3 I-1; I-1; I-3-3 I-1; I-1; I-3-3	0-1; 1-2-3 0-1; 0-2; 2-2-3 0-1; 0-2; 2-2-3 0-1; 0-2; 2-2-3

General morphological resemblance of the buccal appendages among species of *Mastigodiaptomus* was observed in the antenna, mandible, maxillule, maxilla and maxilliped among all the species described here. These resemblances have been described before, supported by the number of segments and the general micro-structure of these appendages (Suárez-Morales & Elías-Gutiérrez, 2000; Gutiérrez-Aguirre, Cervantes-Martínez & Elías-Gutiérrez, 2014; Gutiérrez-Aguirre & Cervantes-Martínez, 2016; Mercado-Salas et al., 2018).

However, after the analysis of the same manuscripts previously mentioned and the biological material considered here, we found that the number of setae on the medial and terminal lobes of Enp2 of the antenna, the number of setae on the maxillule praecoxal arthrite, and the number of setae on the maxillar endopodites and maxillar praecoxal lobes show slight differences between the species of this genus. All these findings are described in Supplemental File 3, and only the differential morphological features of the antenna, maxillule, and maxilla are mentioned in the amended descriptions or diagnosis in the next section.

In the systematics section, a detailed, descriptive morphological analysis of the adult females and males of the new species is presented, in addition to an amended diagnosis of *M. nesus* and a description of morphological variations of *M. patzcuarensis*.

## Systematics

Order Calanoida G.O. Sars, 1903

Family Diaptomidae Baird, 1850

Subfamily Diaptominae Kiefer, 1932

Genus *Mastigodiaptomus* Light, 1939

*Mastigodiaptomus nesus* Bowman, 1986

Figures 1–2

**Figure 1:**
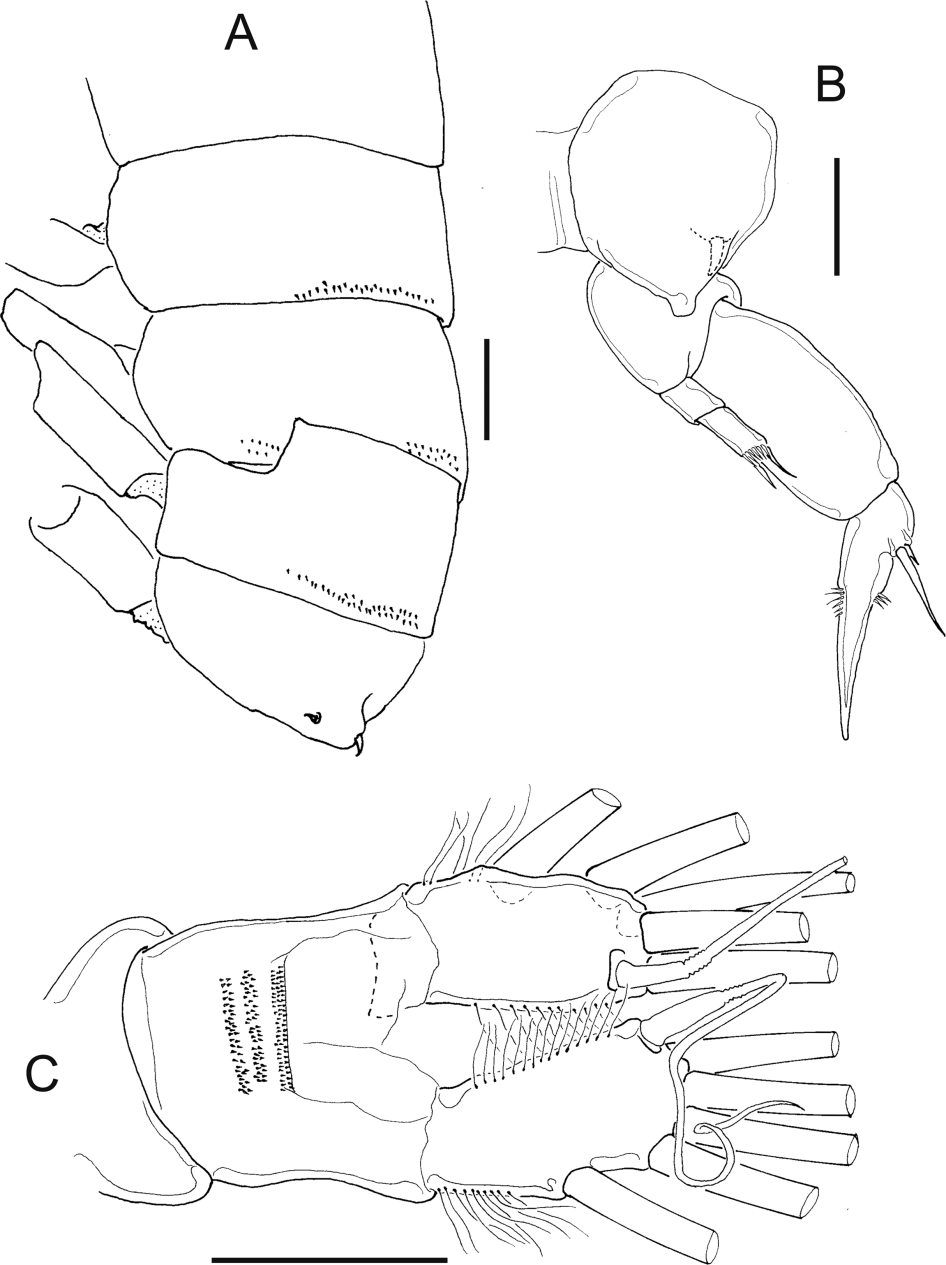
*Mastigodiaptomus nesus* Bowman (1986), female, paratype USNM 216167. (A) Second to fifth prosomites, lateral. (B) Fifth leg. (C) Anal somite and caudal rami, dorsal. Scale bars = 50 µm.

**Figure 2:**
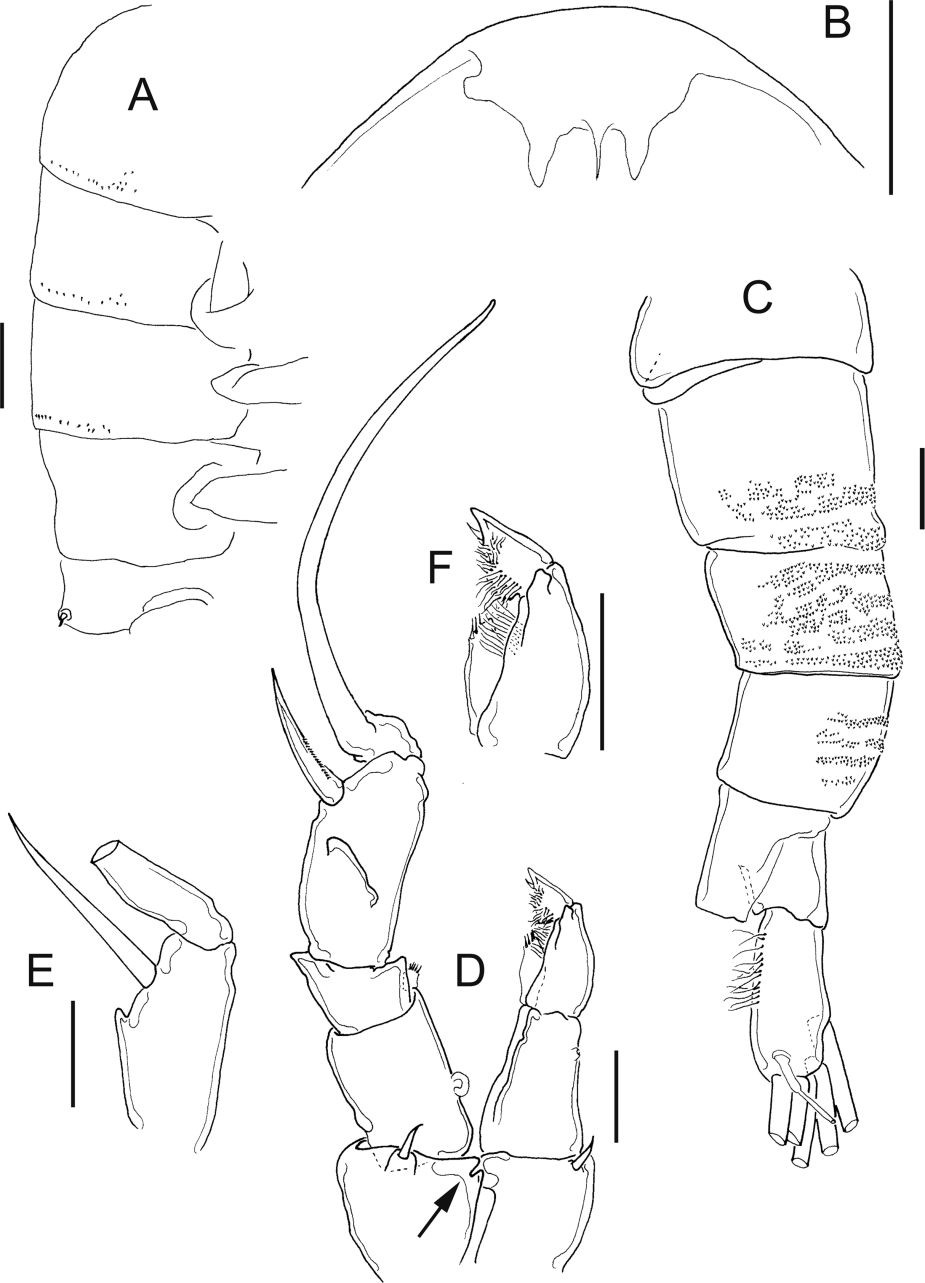
*Mastigodiaptomus nesus* Bowman (1986), male, paratype USNM 216167. (A) First to fifth prosomites, lateral. (B) Rostrum, frontal. (C) Urosomite, dorsal. (D) Fifth leg, caudal, medial lobes arrowed. (E) Fifth leg, right Exp2, lateral. (F) Fifth leg, left Enp, Exp1, and Exp2, caudal. Scale bars = 50 µm.

*Mastigodiaptomus nesus* Bowman, 1986: 237–242; figs. 3, 4

*Mastigodiaptomus nesus* Suárez-Morales et al., 1996: 104; figs. 20a, b; 21a, b, e, h; 22a-c

*Mastigodiaptomus nesus* Cervantes-Martínez et al., 2005: 97–99

*Mastigodiaptomus nesus* Brandorff, 2012: 192, 195; fig. 2

**Material:** Five adult females and five adult males from San Salvador Island, USNM 216168 (paratypes; 24°01′ N; 74°51′ W).

**Additional material:** Ten adult females and 10 adult males preserved in 96% ethanol with a drop of glycerine from each of the following systems: Minicenote 19.IV.2004 (ECO-CH-Z-10312) (19° 36′ 24.8″ N; 87° 59′ 18.8″ W) and El Padre 20.VII.2010 (ECO-CH-Z-10313) (19° 36′ 24.0″ N; 87° 59′ 15.4″ W). One adult female on a semi-permanent slide (ECOCH-Z-10314) and one dissected adult male on a semi-permanent slide (ECOCH-Z-10315) from Laguna Cobá, collected 10.IX.2007 (20° 29′ 32.9″ N; 87° 44′ 15.3″ W).

**Female:** 1.44–1.54 mm body length, including the caudal rami. Prosomites 2–4 with tiny spines on distal latero-dorsal surfaces (Fig. 1A). Twenty-five-segmented antennules, each segment armed with setae, spines, modified setae, or aesthetascs in the following order: (1) 1ms+1ae; (2) 3ms+1ae; (3) 1ms+1ae; (4) 1ms; (5) 1ms+1ae; (6) 1ms; (7) 1ms+1ae; (8) 1ms+1sp; (9) 2ms+1ae; (10) 1ms; (11) 2ms; (12) 1ms+1ae+1sp; (13) 1ms; (14) 1ms+1ae; (15) 1ms; (16) 1s+1ae; (17) 1ms; (18) 1s; (19) 1ms; (20) 1ms; (21) 1s; (22) 1ms+1s; (23) 1ms+1s; (24) 2s; (25) 1ms+3s+1ae.

Antenna with 2-segmented Enp, Enp2 with two lobes: medial (inner) lobe with 8 setae, terminal (outer) lobe with 7 setae. Praecoxal arthrite of maxillule with 8 acute setae on frontal group (+4 setae on caudal group).

Two praecoxal lobes on maxilla; first lobe with 5 setae, second lobe with 3 setae. Three-segmented maxillar Enp, with 1, 2 and 3 setae respectively.

Fifth leg with 2-segmented Enp, with two long apical setae and short setules diagonally arranged. On average, Exp1P5 is 1.8 times longer than EnpP5 (range = 1.6–2.1) (Fig. 1B).

Genital double somite slightly longer than wide, bulbous, and asymmetrical: right spine more proximal and larger than the left spine. Parallel rows of tiny spines on anal dorsal plate; hair-like setae along lateral and medial margins of the caudal rami (Fig. 1C).

**Male:** 1.2–1.4 mm body length, including the caudal rami (average = 1.3 mm). Cuticular surface of prosomites 1, 2 and 3 with tiny setules on the latero-distal margins (Fig. 2A). Rostrum with spines 2.2 ± 0.1 times longer than wide (Fig. 2B). Right geniculate antennule 22-segmented, with a spiniform process on segments 10, 11 and 13 to 16. The tip of the spine on segment 10 reaching the distal margin of the bearing segment. Antepenultimate antennular segment with a hook-like projection. Each segment armed with setae, spines, spiniform process, modified setae, or aesthetascs in the following order: (1) 1ms+1ae; (2) 3ms+1ae; (3) 1ms+1ae; (4) 1ms; (5) 1ms+1ae; (6) 1ms; (7) 1ms+1ae; (8) 1ms+1sp; (9) 2ms+1ae; (10) 1ms+1sps; (11) 1ms+1sps; (12) 1ms+1ae+1sp; (13) 1ms+1sps; (14) 2ms+1ae+1sps; (15) 2ms+1ae+1sps; (16) 2ms+1ae+1sps; (17) 1ms; (18) 1ms; (19) 1ms+1ae; (20) 1ms+1ae+1s; (21) 2s; (22) 4s. Left male antennule, antenna, maxillule and maxilla similar to the female.

Urosomites 2–4 with parallel rows of tiny spines on dorsal surfaces; caudal rami pilose medially (Fig. 2C).

Coxal segments of male P5 with one long spine each; right coxa bilobed medially (arrow in Fig. 2D); right basipodite rectangular, without proximal-medial protrusion, with one rounded hyaline membrane on the medial margin. Right Exp1 is 2 times longer than the right Enp (Fig. 2D). Straight ridge on right Exp2 laterally projected (Fig. 2E). Distal aculeus 0.7 times the length of the right Exp2.

Left Exp1 of male P5 is 1.7 times longer than the left Enp, and left Exp2 is distally attenuated, almost triangular in shape, hairy (Fig. 2F).

It is noteworthy that females and males from El Padre, Minicenote, and Laguna Cobá (southeastern Mexico) are shorter in body length (0.90–1.0 mm in both sexes) than the paratypes, these latter with a body length from 1.44 to 1.54 mm in females and 1.30 to 1.44 in males (Bowman, 1986).

**Distribution:** Neotropical. Caribbean Islands (Bowman, 1986), Yucatan Peninsula (Mexico) states Quintana Roo, Yucatán, Campeche and Belize (Brandorff, 2012).

*Mastigodiaptomus alexei* sp. n. Elías-Gutiérrez

urn:lsid:zoobank.org:act:FFF6D019-9464-41E5-A992-E0C45B109CE7

Figures 3–6

**Figure 3:**
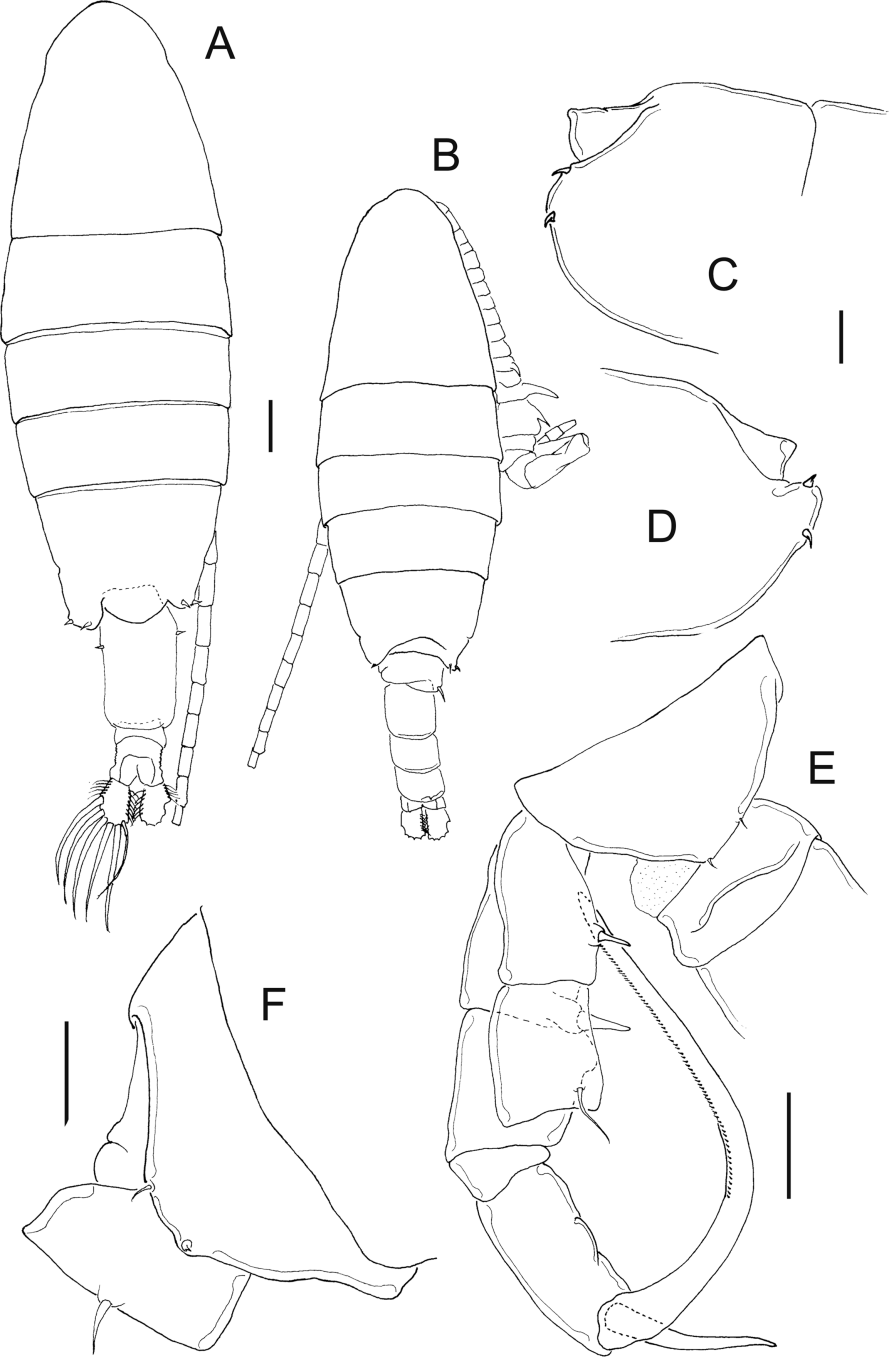
*Mastigodiaptomus alexei* sp. n., adult female, holotype (A, C and D) and adult male, allotype (B, E and F). (A) Habitus, dorsal. (B) Habitus, dorsal. (C) Right wing of last prosomite. (D) Left wing of last prosomite. (E) Left wing of last prosomite, fifth leg (left Exp omitted) and first urosomite, lateral. (F) Right wing of last prosomite and first urosomite, lateral. Scale bars = 100 µm in (A) and (B); 50 µm in (C)–(F).

**Figure 4:**
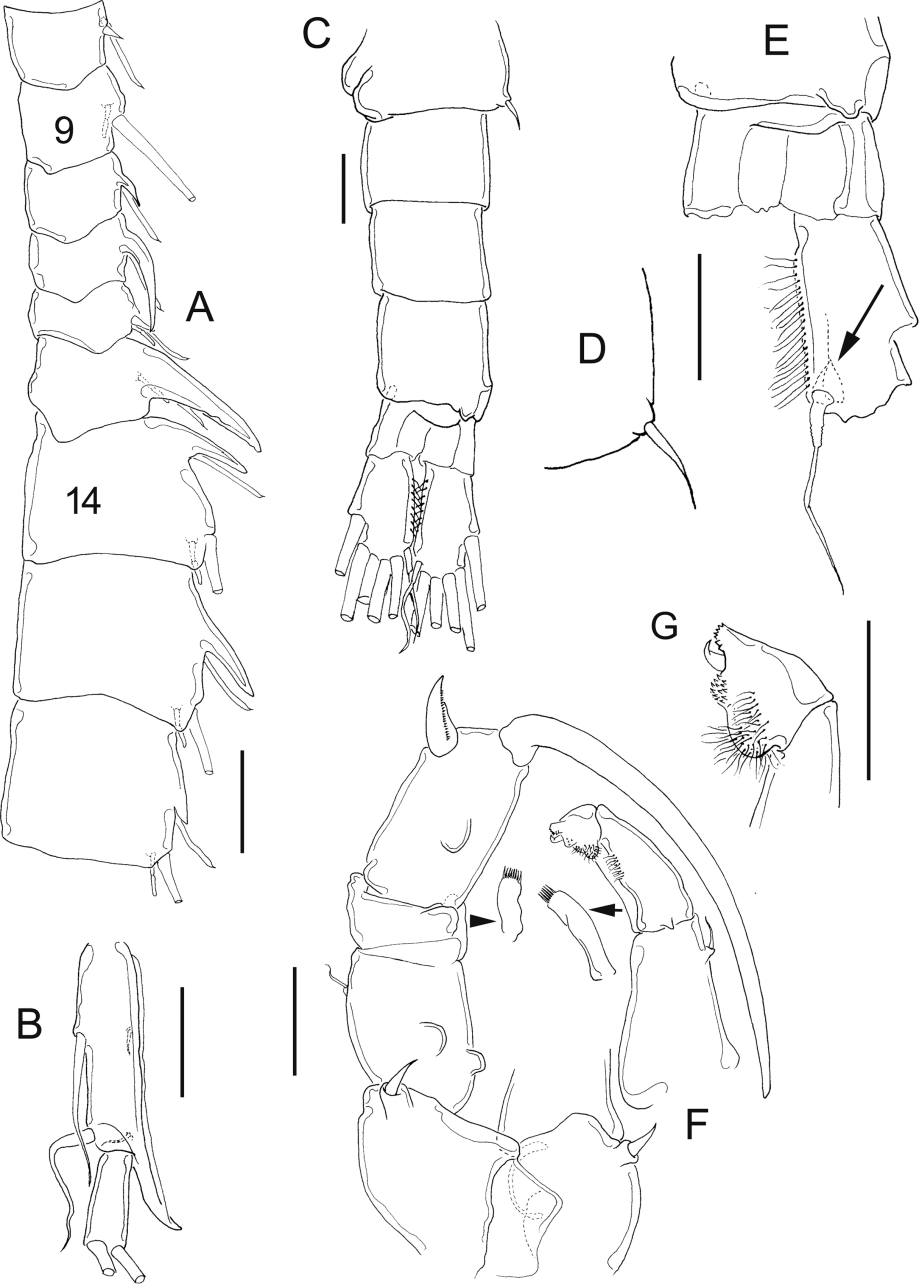
*Mastigodiaptomus alexei* sp. n., allotype. (A) Right antennule, segments 8–16. (B) Right antennule, segments 20, 21. (C) Urosomite, dorsal. (D) Detail of the right spine on the first urosomite. (E) Preanal somite, anal somite, and right furcal ramus, dorsal (ventral hump arrowed). (F) Fifth leg, caudal (endopodites and left rami separated; the length of the aculeus is due to its oblique position). (G) Detail of left Exp2. Scale bars = 50 µm.

**Figure 5:**
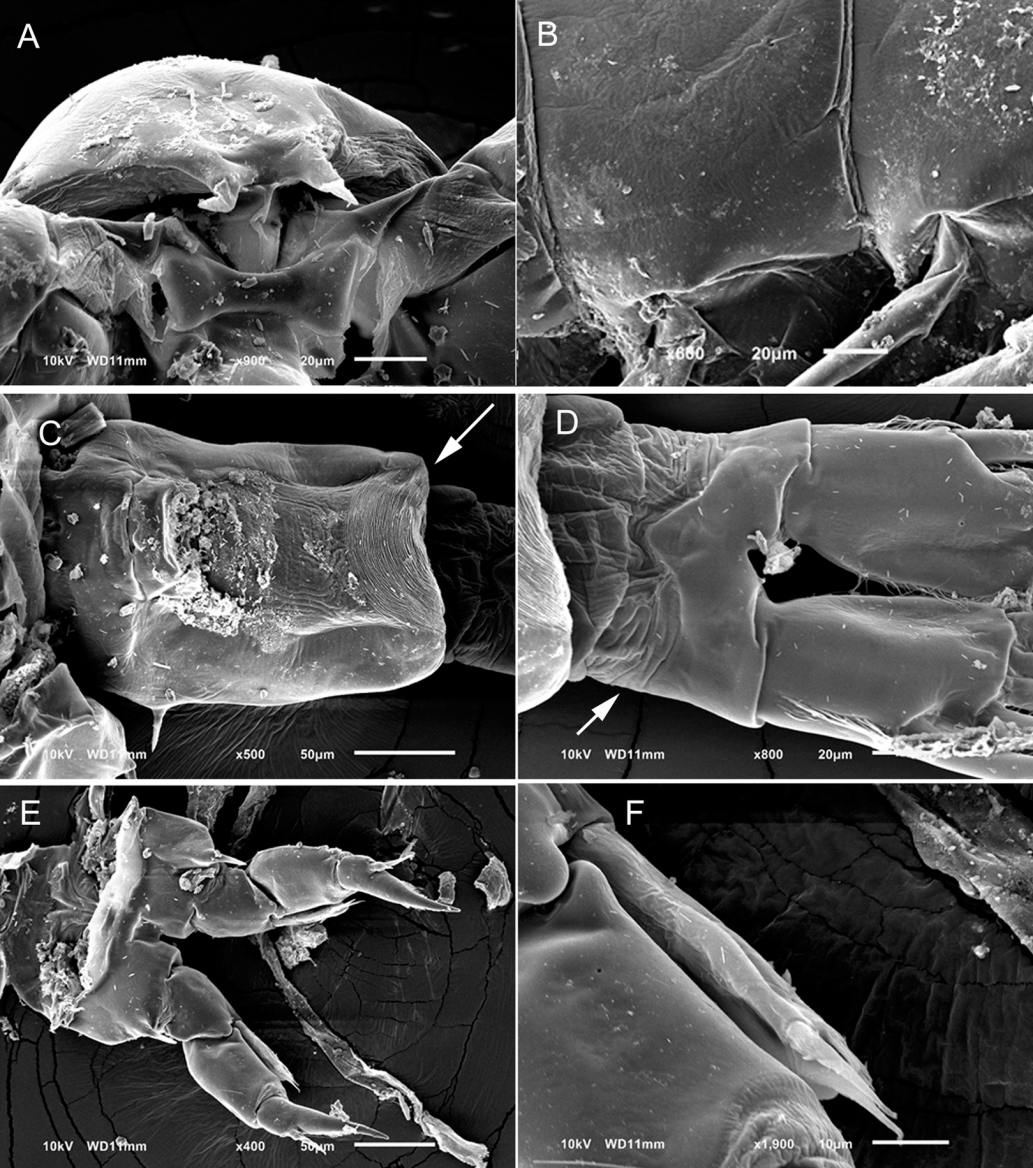
*Mastigodiaptomus alexei* sp. n. adult female. (A) Rostrum and mouth region. (B) Detail of second and third prosomites, lateral. (C) Genital somite, ventral scars arrowed. (D) Anal somite, ventro-lateral scars arrowed. (E) Fifth leg, caudal. (F) Fifth leg, detail of Enp.

**Figure 6:**
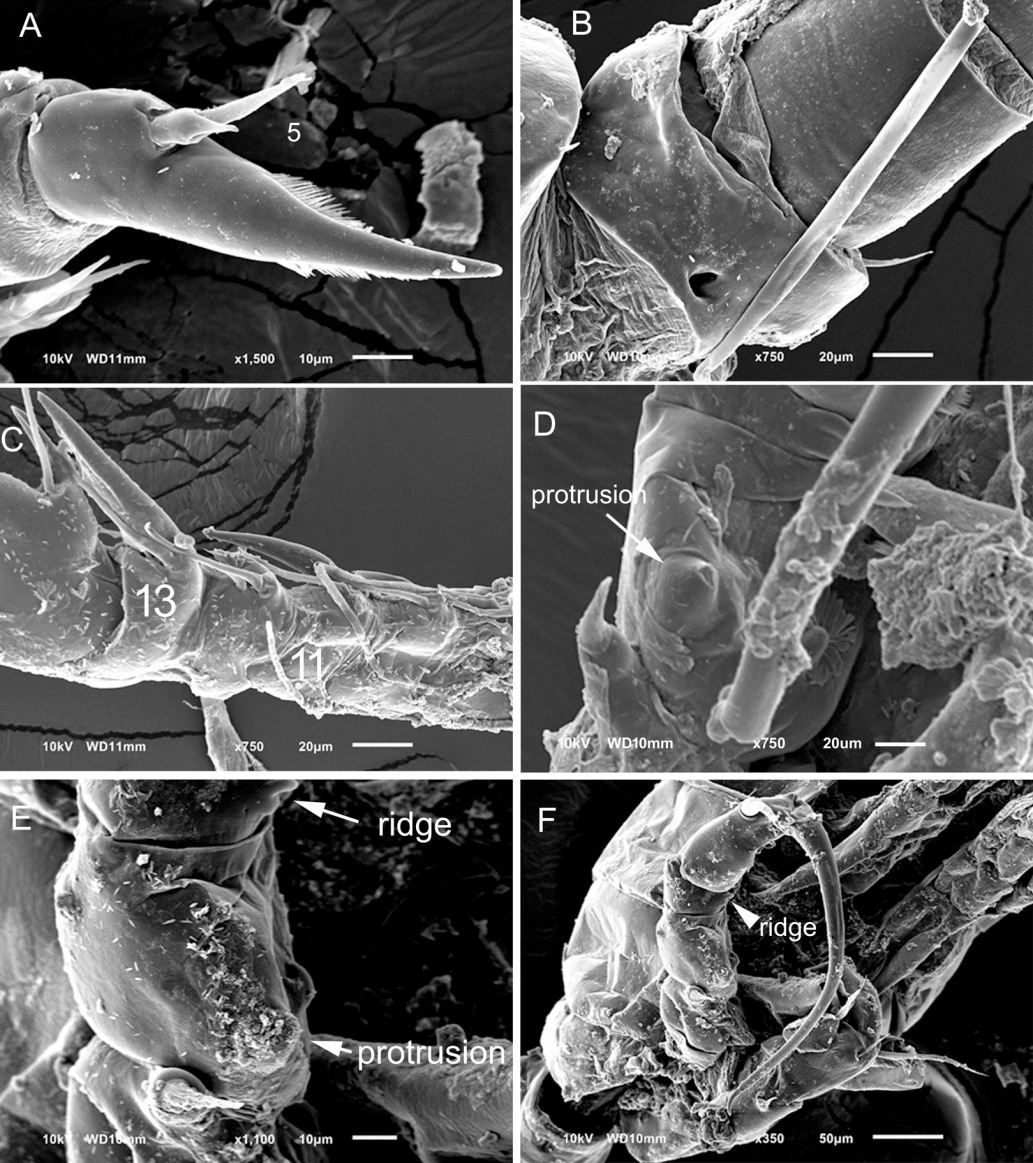
*Mastigodiaptomus alexei* sp. n. adult female (A) and adult male (B–F). (A) Fifth leg, detail of Exp2 and Exp3. (B) First and second urosomites, ventral. (C) Right antennule, segments 9–13. (D) Right fifth leg, detail of Bsp. (E) Right fifth leg, detail of Bsp and Exp1. (F) Fifth leg, caudal.

**Holotype:** One adult female preserved in 96% ethanol with a drop of glycerine (ECOCH-Z-10316).

**Allotype:** One adult male dissected on one semi-permanent slide (ECOCH-Z-10317).

**Paratypes:** Two adult females and 20 adult males preserved in 96% ethanol and one drop of glycerine (ECOCH-Z-10318).

**Type locality:** Wetland near Camalote, Veracruz, México. Collected 10.IX.2005 (22° 0′ 43.2″ N; 98° 13′ 37.2″ W).

**Etymology:** The species is dedicated to the son of MEG, who has assisted in field collections since the age of 5 years old.

**Additional material:** Two adult females and males for genetic analysis from wetland near Camalote, Veracruz, Mexico. Three adult females and three adult males for SEM analysis from the same place.

**Diagnosis:** Female prosomites and urosomites dorsal and laterally smooth, genital somite slightly asymmetric, lateral borders almost parallel, antennules reaching the furcal rami, and furcal rami with long setules along both lateral and medial margins (Fig. 3A). Lateral wings of the last prosomite projected, with two spines on each side; left wing more distally projected than the right wing (Figs. 3C and 3D). Antenna with 2-segmented Enp, Enp2 with two lobes: medial lobe with 9 setae, terminal lobe with 7 setae. Praecoxal arthrite of maxillule with 9 acute setae on frontal group (+4 setae on caudal group). Two praecoxal lobes on maxilla; first lobe with 4 setae, second lobe with 3 setae. Three-segmented maxillar Enp, with 1, 1 and 3 setae respectively. Similar to that in female, the males bear two spines on each lateral wing of the last prosomite (Figs. 3E and 3F), and the antenna, maxillule and maxilla as previously described for females. First male urosomite with a long spine on the right side, directed distally (Fig. 3F). Right Exp2 of fifth male leg with one short distal aculeus, one sclerotisation on the caudal surface, and one long distal spine. This distal spine, slightly longer than the length of the right leg, is curved and directed towards the distal region (Fig. 3E). Left Exp2 of fifth male leg robust, triangular, and with delicate teeth apically (Fig. 4G). Right male antennule 22-segmented, with a spiniform process on segments 10, 11 and 13 to 16 (Fig. 4A). Furcal rami of males with long setae along the medial margin (Fig. 4E).

**Female:** 1.4–1.5 mm body length, including the caudal rami. Rostrum with pointed spines (Fig. 5A). Latero-ventral and dorsal surfaces of the prosomites smooth, without ornamentation (Fig. 5B). Holotype and paratypes without dorsal process on the last prosomite. Twenty-five-segmented antennules, each segment armed as follows: (1) 1ms+1ae; (2) 3ms+1ae; (3) 1ms+1ae; (4) 1ms; (5) 1ms+1ae; (6) 1ms; (7) 1ms+1ae; (8) 1ms+1sp; (9) 2ms+1ae; (10) 1ms; (11) 2ms; (12) 1ms+1ae+1sp; (13) 1ms; (14) 1ms+1ae; (15) 1ms; (16) 1ms+1ae; (17) 1ms; (18) 1ms; (19) 1ms+1ae; (20) 1ms; (21) 1ms; (22) 1ms+1s; (23) 1ms+1s; (24) 1ms+1s; (25) 4s+1ae.

The second and third prosomites are the widest regions of the body; the left wing of the last segment is more projected towards the distal region of the body than the right wing; antennule, reaching the distal margin of the caudal rami (Fig. 3A).

Right and left wings of the last prosomite with dorsal and ventral spines directed laterally (Figs. 3C and 3D). Genital somite asymmetric, 1.6–1.8 times longer than wide; left margin almost straight, with a strong spine placed more distally than the right spine, the latter spine inserted on the slightly protruding right margin (Figs. 3A and 5C). Distal, ventral surface of the genital double somite with deep scars on the cuticle, which are arranged in a parallel pattern (arrow, Fig. 5C). Anal somite with lateral folds (arrow, Fig. 5D) and hair-like setae along the lateral and medial margins of the caudal rami (Fig. 5D).

**Fifth leg:** Coxa with left and right lateral spines (Fig. 5E); 2-segmented Enp, which bears 2 long setae and a row of short setules apically (Fig. 5F). The Enp is 0.8–0.9 times the length of the inner margin of Exp1. Second exopodite with spines along the margins and one lateral, short spine placed next to Exp3. The latter is separated and bears one long plus one short spine (Fig. 6A).

**Male:** 1.1–1.3 mm body length. As in females, the cuticular surfaces of the pro- and urosomites are smooth (Fig. 6B). Left male antennule as in female; right geniculate antennule 22-segmented, with a spiniform process on segments 10, 11 and 13–16. Spiniform process on segment 10 reaching the distal margin of the bearing segment. Spiniform process on segment 11 reaching the distal margin of segment 12 (Figs. 4A and 6C).

Segments of right geniculate A1 armed as follows: (1) 1ms+1ae; (2) 3ms+1ae; (3) 1ms+1ae; (4) 1ms; (5) 1ms+1ae; (6) 1ms; (7) 1ms+1ae; (8) 1ms+1sp; (9) 1ms+1ae; (10) 1ms+1sps, (11) 1ms+1sps; (12) 1ms+1ae+1sp; (13) 1ms+1ae+1sps; (14) 2ms+1ae+1sps; (15) 2ms+1ae+1sps; (16) 2ms+1ae+1sps; (17) 1ms; (18) 0; (19) 1ms+1ae; (20) 1ms+1ae+1s; (21) 2s; (22) 4s (Fig. 4A). Spiniform process of antennular segments 15 and 16 projecting from the mid-length of the bearing segment.

Antepenultimate antennular segment with a hook-like projection that reaches the distal third of the penultimate antennular segment (Fig. 4B) and with a lateral hyaline membrane.

Left wing of the last prosomite with one tiny spine and one tiny setule in the ventral and dorsal positions, respectively (Fig. 3E). The spine and seta on the right wing of the last prosomite (Fig. 3F) are larger than those on the left one.

First urosomite with one spine on the right margin, this spine thinner distally (Figs. 4C and 4D); pre-anal somite asymmetric: right margin larger than left margin and with angled protuberances (Fig. 4E), caudal rami pilose medially; right ramus with a bulbous hump on the ventral surface (arrow, Fig. 4E).

Right fifth leg, caudal view: Coxopodite with a large lateral spine, this segment strongly sclerotised distally and bent to the left. Basipodite folded distally, with a lateral short seta and one rounded hyaline membrane on the medial margin; one protrusion present on the mid-caudal face of the basipodite, which is very high (Figs. 4F, 6D and 6E). Exp1 with a ridge on the medial margin (arrowed in Figs. 6E and 6F) and with one angled lamella laterally. Endopodite is twice the length of the medial margin of Exp1.

Exp2 is 1.7–1.8 times longer than wide, with crescent-shaped sclerotisation; this exopodal segment is 1.1–1.2 times longer than the aculeus. This aculeus is placed distally and directed posteriorly, as is the strongly curved distal spine (Fig. 3E). The distal spine is 1.1–1.2 times longer than the entire right leg.

Left fifth leg (Fig. 4F): Coxopodite with one long, lateral spine and bulbose medially. Basipodite proximally bulbose, with one small lateral setule. Left Exp1 slightly longer than Enp (1.3:1.0) and 2.6–2.7 times longer than Exp2. Exp2 lobed: proximal lobe pilose, distal lobe with spinules. Distally, Exp2 with a serrated margin flanked by a curved seta (Fig. 4G).

**Distribution:** Mexico, found only in a wetland near Camalote, Veracruz (the type locality).

*Mastigodiaptomus ha* sp. n. Cervantes-Martínez

urn:lsid:zoobank.org:act:0974ECE1-BF2A-4844-A5EA-4880CC957D1B

Figures 7–10

**Figure 7:**
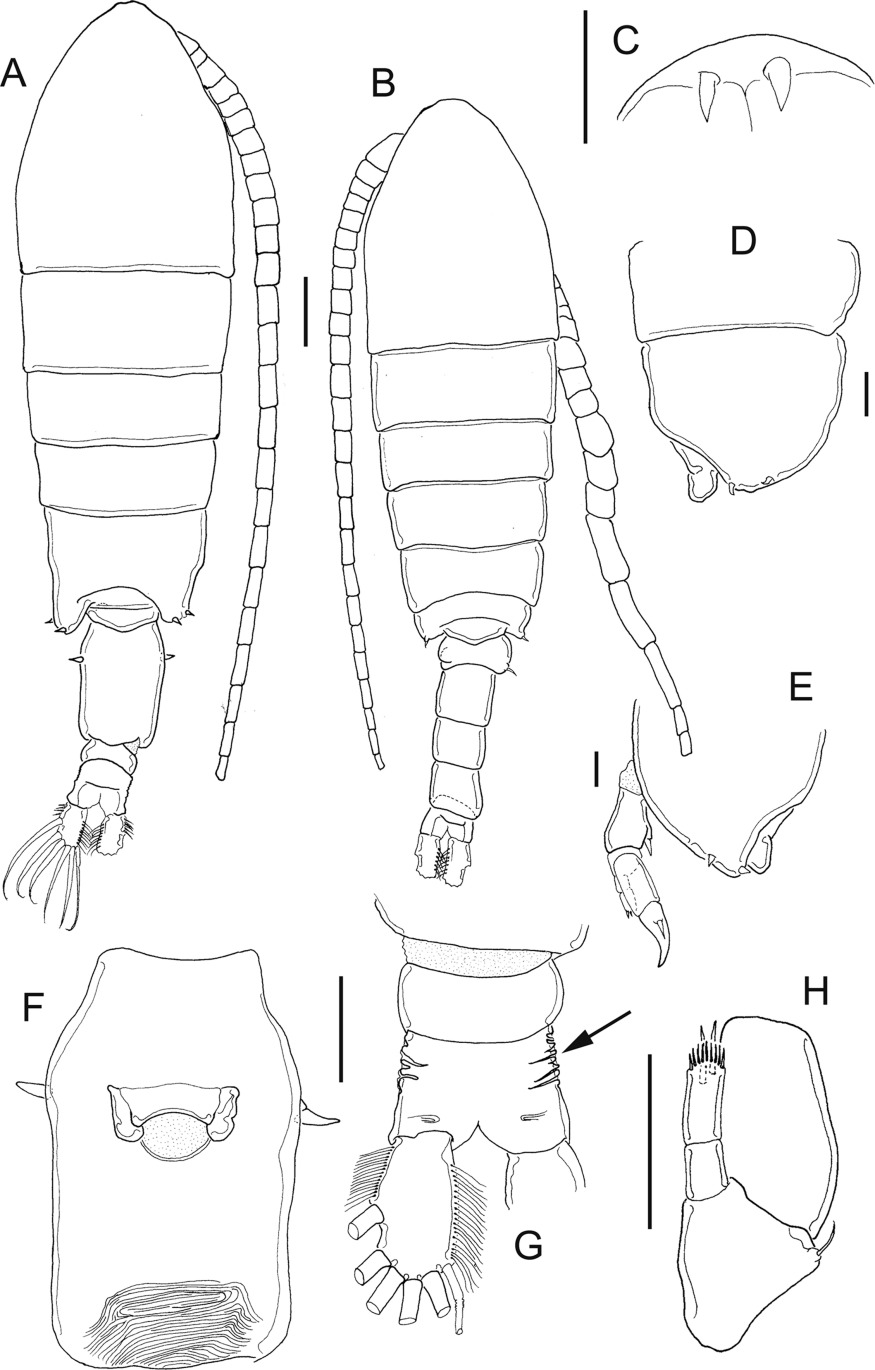
*Mastigodiaptomus ha* sp. n., adult female, holotype (A and C–H) and adult male, allotype (B). (A) Habitus, dorsal. (B) Habitus, dorsal. (C) Rostrum. (D) Right wing of last prosomite. (E) Left wing of last prosomite. (F) Genital somite, ventral. (G) Pre-anal somite, anal somite, and caudal ramus, ventral (lateral folds arrowed in anal somite). (H) Fifth leg, detail of basipodite, Enp and Exp1. Scale bars = 100 µm in (A) and (B); 50 µm in (C)–(H).

**Figure 8:**
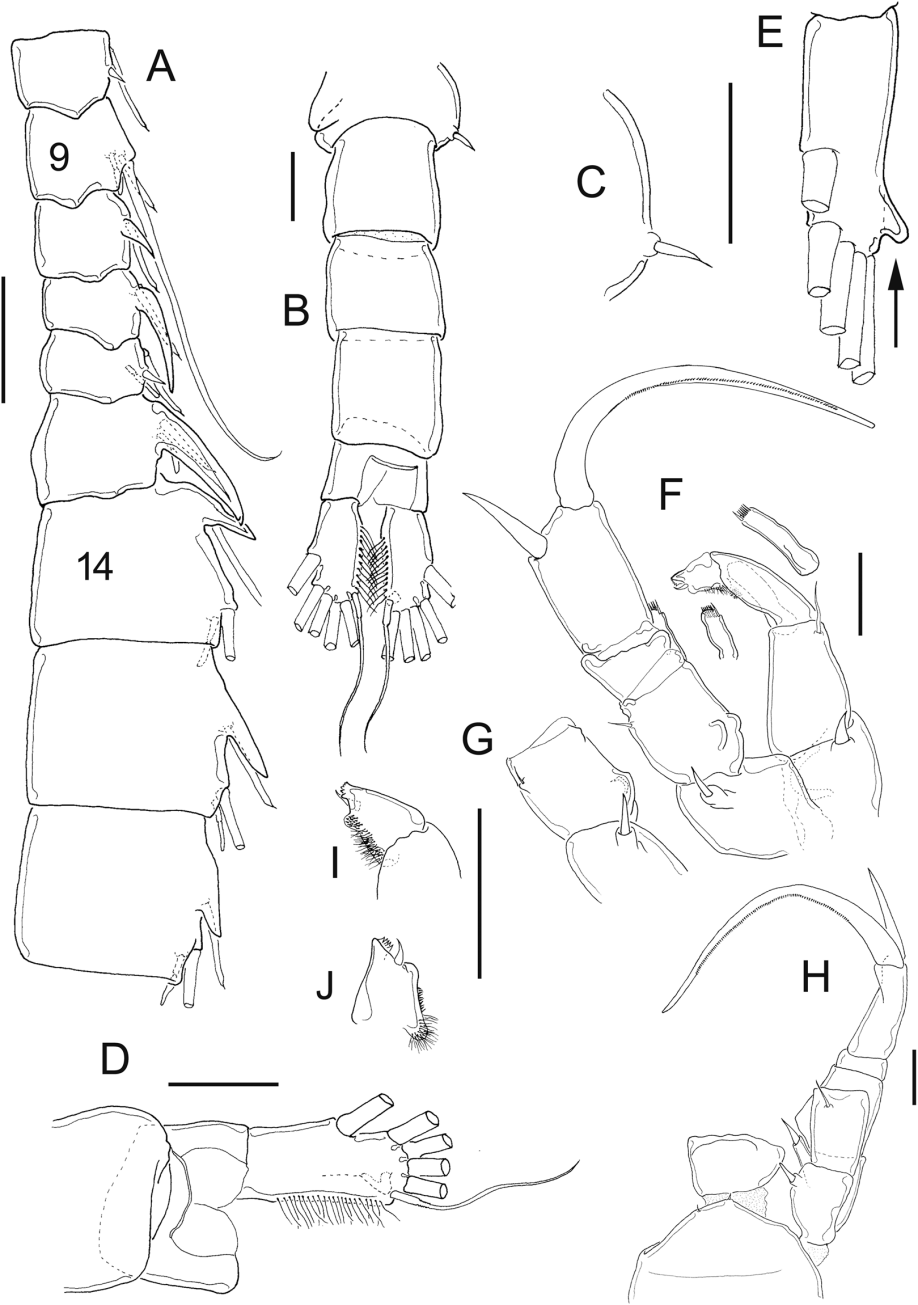
*Mastigodiaptomus ha* sp. n., allotype. (A) Right antennule, segments 8–16. (B) Urosomite, dorsal. (C) Detail of the right spine on the first urosomite. (D) Pre-anal somite, anal somite, and right caudal ramus, dorsal. (E) Right caudal ramus, lateral (bulbous hump arrowed). (F) Fifth leg, caudal (note the Enps are separated). (G) Detail of right coxo- and basipodite, lateral. (H) Left wing of last prosomite, fifth leg (left Exp omitted) and first urosomite, lateral. (I) Detail of left Exp2, caudal. (J) Detail of left Exp2, frontal. Scale bars = 50 µm.

**Figure 9:**
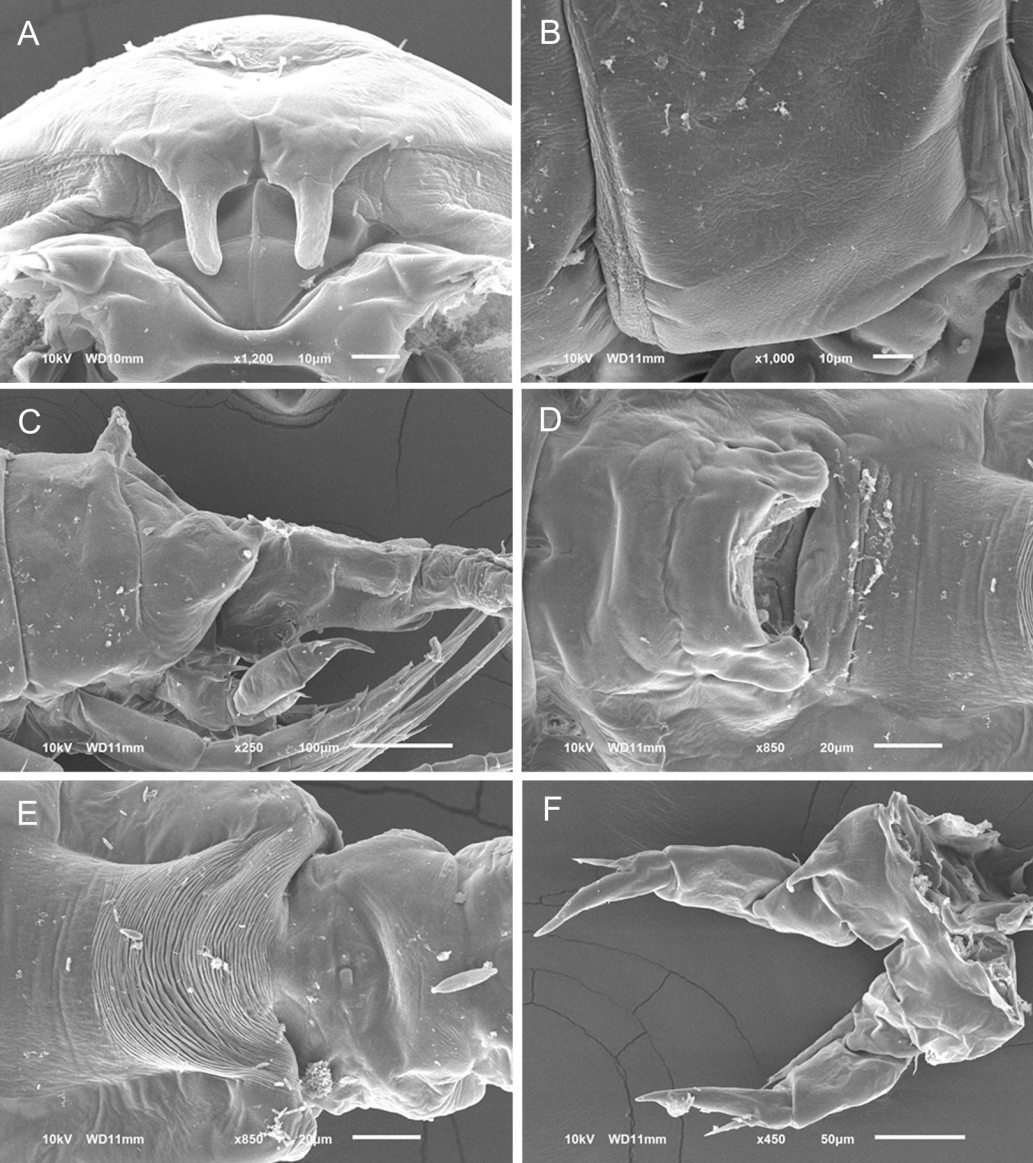
*Mastigodiaptomus ha* sp. n., adult female. (A) Rostrum. (B) Third prosomite, lateral. (C) Last prosomites and urosomites, lateral. (D) Genital field, ventral. (E) Genital somite, and pre-anal somite, ventral. (F) Fifth leg, caudal.

**Figure 10:**
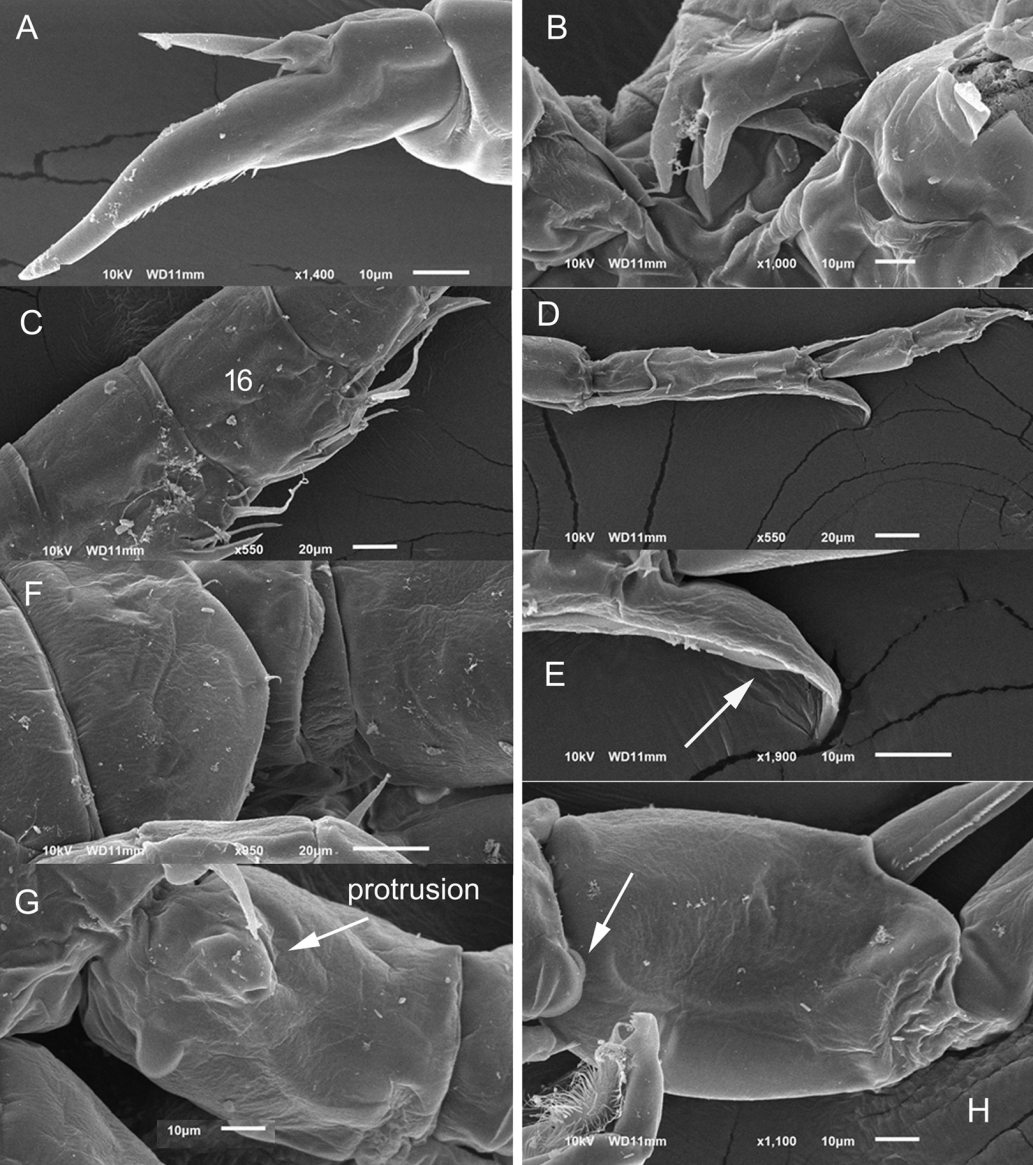
*Mastigodiaptomus ha* sp. n., adult female (A) and adult male (B–H). (A) Fifth leg, Exp2 and Exp3. (B) Rostrum. (C) Right antennule, segments 15–17. (D) Right antennule, segments 20–22. (E) Right antennule, detail of projection of segment 20 arrowed. (F) Left wing, lateral. (G) Fifth leg, detail of right basipodite, caudal (low protrusion arrowed). (H) Fifth leg, detail of right Exp1 (rounded lamella arrowed), Exp2, and left Exp2, caudal.

? *Mastigodiaptomus texensis* Suárez-Morales et al., 1996: 104–106; figs. 20e, f; 21g, j; 22g, i; 87c.

**Holotype:** One adult female dissected on a semi-permanent slide (ECOCH-Z-10319).

**Allotype:** One adult male dissected on a semi-permanent slide (ECOCH-Z-10320).

**Paratypes:** 20 adult females and 20 males preserved in 96% ethanol and one drop of glycerine (ECOCH-Z-10321).

**Type locality:** Cenote 7 Bocas, Quintana Roo, México. Collected 30.VII.2010 (20° 52′36″ N; 87° 02′37″ W).

**Etymology:** The species name is a noun in apposition, and the term ‘ha’ means ‘water’ in the Mayan language.

**Additional material:** Five adult females and five adult males for genetic analysis from Verde Lucero (20° 52′09.57″ N; 87° 04′37.52″ W) and 7 Bocas. Five adult females and five adult males for SEM analysis from Cenote 7 Bocas. Three adult females and three adult males preserved in 90% ethanol and one drop of glycerine; Cenote Boca del Puma Abierto (20° 52′179″ N; 87° 03′18″ W); collected 30.VI.2010 (ECOCH-Z-10326). Fifteen adult females and 15 adult males preserved in 90% ethanol and one drop of glycerine; Cenote Verde Lucero; collected 30.VI.2010 (ECOCH-Z-10327).

**Diagnosis:** Female with short antennules, reaching the second urosomite; fifth prosomite with or without a dorsal projection; and the left wing of the fifth prosomite slightly projected distally (Fig. 7A). Antenna with 2-segmented Enp, Enp2 with two lobes: medial lobe with 9 setae, terminal lobe with 7 setae. Praecoxal arthrite of maxillule with 11 acute setae on frontal group (+4 setae on caudal group). Two praecoxal lobes on maxilla; first lobe with 4 setae, second lobe with 3 setae. Three-segmented maxillar Enp, with 1, 2 and 3 setae respectively. Genital double somite clearly longer than wide, almost symmetric, but the right lateral spine is inserted more proximally than the left spine (Fig. 7F). Wrinkles on the ventral surface of the genital double somite and anal somite (Figs. 7F and 7G). Caudal rami with hairy lateral and medial margins (Fig. 7G). Endopod of fifth leg two-segmented and slightly shorter than the Exp1 of the fifth leg (Fig. 7H). The right and left prosomal wings of males with spines; first urosomite with a lateral, strong spine on the right margin (Fig. 7B); right male antennule with a weak, short spine on segment 8 but a long spinal process on segments 10, 11 and 13–16 (Fig. 8A). Antenna, maxillule and maxilla as described for females. Pre-anal segment with a rounded, right protrusion and caudal rami with the medial margin hairy in males (Figs. 8B and 8D). Right fifth male leg with a basipodite bearing one basal protrusion and one basal hyaline membrane, right Exp2 almost rectangular, with frontal and caudal surfaces smooth and bearing one straight distal aculeus (Figs. 8F and 8G). Long left Exp1, is 2.6 times longer than the left Exp2 (Fig. 8F).

**Female:** 1.2–1.3 mm body length, including the caudal rami. Holotype without a dorsal process on the last prosomite (Fig. 7A), but in 20% of female paratypes, this process is present (Fig. 9C). The second prosomite is the widest region of the body. In comparison with the right wing, the left wing of the last segment is slightly more projected towards the distal region of the body; antennule reaches the half length of the anal somite (Fig. 7A). Rostrum with pointed (Fig. 7C) or rounded spines (Fig. 9A). Latero-ventral and dorsal surfaces of prosomites smooth, without ornamentation (Fig. 9B).

Antennules 25-segmented, each segment armed as follows: (1) 1ms+1ae; (2) 3ms+1ae; (3) 1ms+1ae; (4) 1ms; (5) 1ms+1ae; (6) 1ms; (7) 1ms+1ae; (8) 1ms+1sp; (9) 2ms+1ae; (10) 1ms; (11) 2ms; (12) 1ms+1ae+1sp; (13) 1ms; (14) 1ms+1ae; (15) 1ms; (16) 1s+1ae; (17) 1ms; (18) 1s; (19) 1ms+1ae; (20) 1ms; (21) 1s; (22) 1ms+1s; (23) 1ms+1s; (24) 2s; (25) 3s+2ae.

Right and left wings of the last prosomite with dorsal and ventral spines directed laterally (Figs. 7D and 7E). Lateral margins of the genital double somite almost parallel, 1.6–1.7 times longer than wide; left margin almost straight, with a strong spine placed slightly more distally than the right spine (Fig. 7A and 7F). Genital field quadrangular (Fig. 9D). Distal, ventral surface of the genital double somite with deep scars on the cuticle curved, parallel, sometimes forming a pattern similar to fingerprints (Figs. 7F and 9E). Anal somite with lateral folds (arrow Fig. 7G) and hair-like setae along the lateral and medial margins of the caudal rami (Fig. 7G).

**Fifth leg:** Coxa with lateral spines (Fig. 9F); 2-segmented Enp, which bears 2 long setae and a row of short setules apically (Fig. 7H). The Enp is 0.8–0.9 times the length of the inner margin of Exp1 (Fig. 7H). Second exopodite with spines along the margins and one short lateral spine placed next to Exp3. The latter is separated and bears one long plus one short spine (Fig. 10A).

**Male:** 1.2–1.3 mm body length. Cuticular surfaces of pro- and urosomites smooth (Fig. 7B). Rostral spines slightly more pointed than in the female (Fig. 10B). Right geniculate antennule 22-segmented, with spiniform process on segments 10, 11 and 13–16 (Fig. 8A). Spiniform process on segment 10 reaching the distal margin of the bearing segment. Spiniform process on segment 11 reaching the distal margin of segment 12 and 1.5 times longer than the width of the bearing segment (Fig. 8A).

Segments of right geniculate A1 armed as follows: (1) 1ms+1ae; (2) 3ms+1ae; (3) 1ms+1ae; (4) 1ms; (5) 1ms+1ae; (6) 1ms; (7) 1ms+1ae; (8) 1ms+1sp; (9) 2ms+1ae; (10) 1ms+1sps, (11) 1ms+1sps; (12) 1ms+1ae+1sp; (13) 1ms+1ae+1sps; (14) 2ms+1ae+1sps; (15) 2ms+1ae+1sps; (16) 2ms+1ae+1sps; (17) 1ms; (18) 1sp; (19) 1ms+1ae; (20) 2s+2ae; (21) 2s; (22) 3s+2ae (Fig. 8A). Left male A1 armed as in female.

Spiniform processes of antennular segments 15 and 16 projecting from the mid-length of the bearing segment and spiniform process of antennular segment 16 similar to a flat, hollow cavity (Fig. 10C). Antepenultimate antennular segment with a hook-like projection that reaches the distal third of the penultimate antennular segment (Fig. 10D) and with a lateral hyaline membrane (Fig. 10E, arrow).

Left wing of the last prosomite with one small spine and one small setule in the ventral and dorsal positions, respectively (Fig. 10F). The spine and seta on the right wing of the last prosomites are larger than those on the left wing.

First urosomite with one spine on the right margin (Figs. 8B and 8C); pre-anal somite with a rounded protrusion on the distal, dorsal right corner, caudal rami pilose medially (Fig. 8D); right ramus with a bulbous hump on the ventral surface (Figs. 8D and 8E).

Right fifth leg, caudal view: Coxopodite with a large lateral spine. Basipodite folded distally, with a lateral short seta and one rounded hyaline membrane on the medial margin; one protrusion present on the mid-caudal face of the basipodite (Fig. 8F), which is low (Fig. 8G, arrow Fig. 10G). Exp1 with rounded lamellae on the medial and lateral margins (Fig. 8F; arrow, Fig. 10H). Endopodite is twice the length of the medial margin of Exp1. Exp2 is 1.6–1.7 times longer than wide, with a smooth surface (Figs. 8F and 10H); this exopodal segment is 1.4–1.5 times longer than the aculeus. This aculeus is placed distally (Fig. 8F), as is the strongly curved distal spine; the distal spine is as long as the entire right leg (Fig. 8H).

Left fifth leg: Coxopodite with one long lateral spine and bulbose medially; basipodite proximally bulbose, with one small lateral setule (Fig. 8F). Left Exp1 slightly longer than Enp (1.3:1.0) and 2.6–2.7 times longer than Exp2. Exp2 lobed: proximal lobe pilose, distal lobe with spinules; distally, this Exp2 with a serrated margin flanked by a curved seta (Figs. 8I, 8J and 10H).

**Distribution:** Mexico, ruta de cenotes (cenote route) Quintana Roo: 7 Bocas (seven mouths), Verde Lucero (green star), and Boca del Puma (panther mouth) sinkholes.

*Mastigodiaptomus cihuatlan* sp. n. Gutiérrez-Aguirre

urn:lsid:zoobank.org:act:CF36F659-886A-4069-BF03-F1C300AE4FB8

Figures 11–13

**Figure 11:**
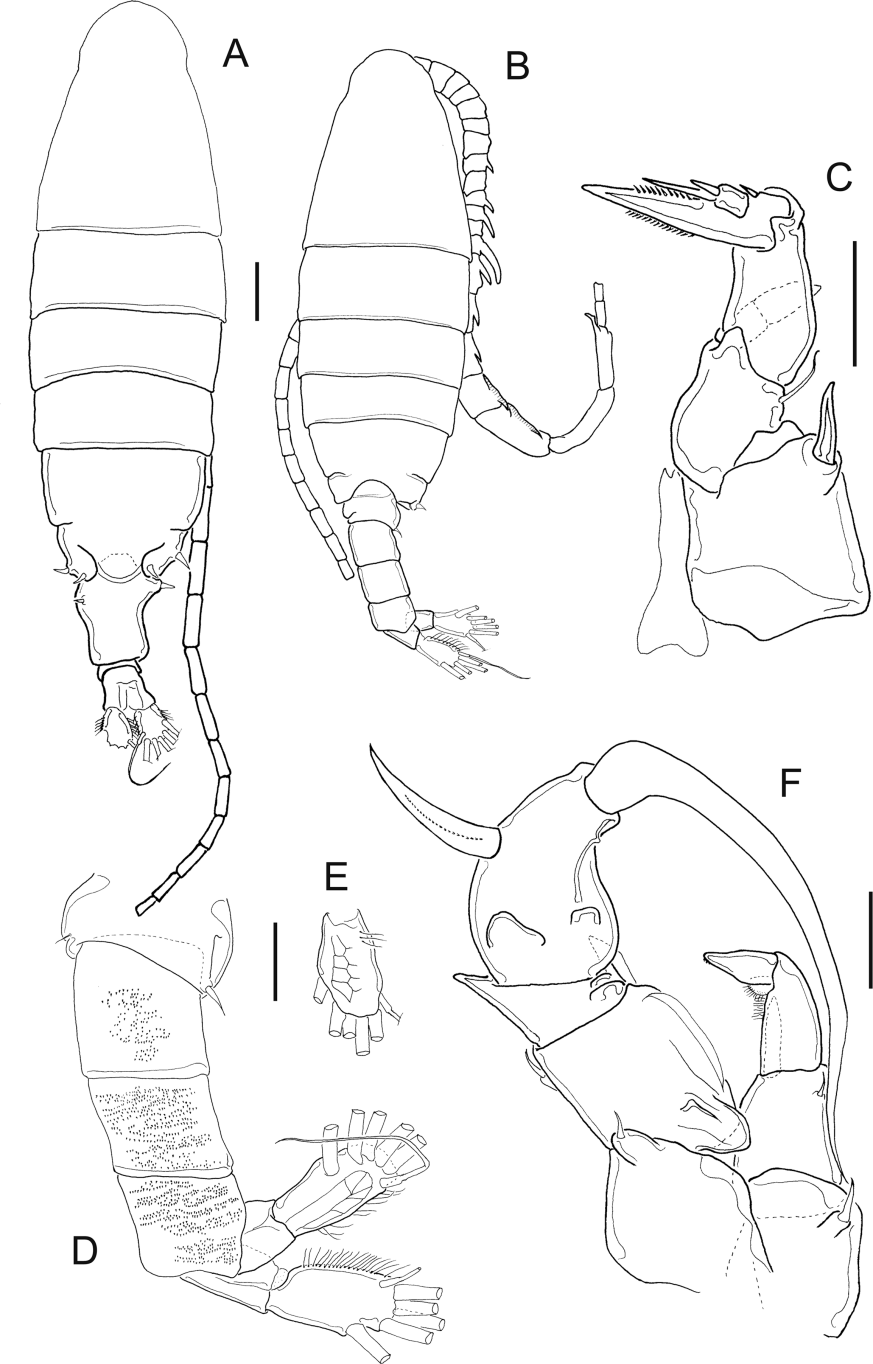
*Mastigodiaptomus cihuatlan* sp. n., adult female, holotype (A and C) and adult male, allotype (B and D–F). (A) Habitus, dorsal. (B) Habitus, dorsal. (C) Fifth leg, caudal. (D) Urosome, dorsal. (E) Right caudal ramus, ventral. (F) Fifth leg, caudal. Scale bars = 100 µm in (A) and (B); 50 µm in (C)–(F).

**Figure 12:**
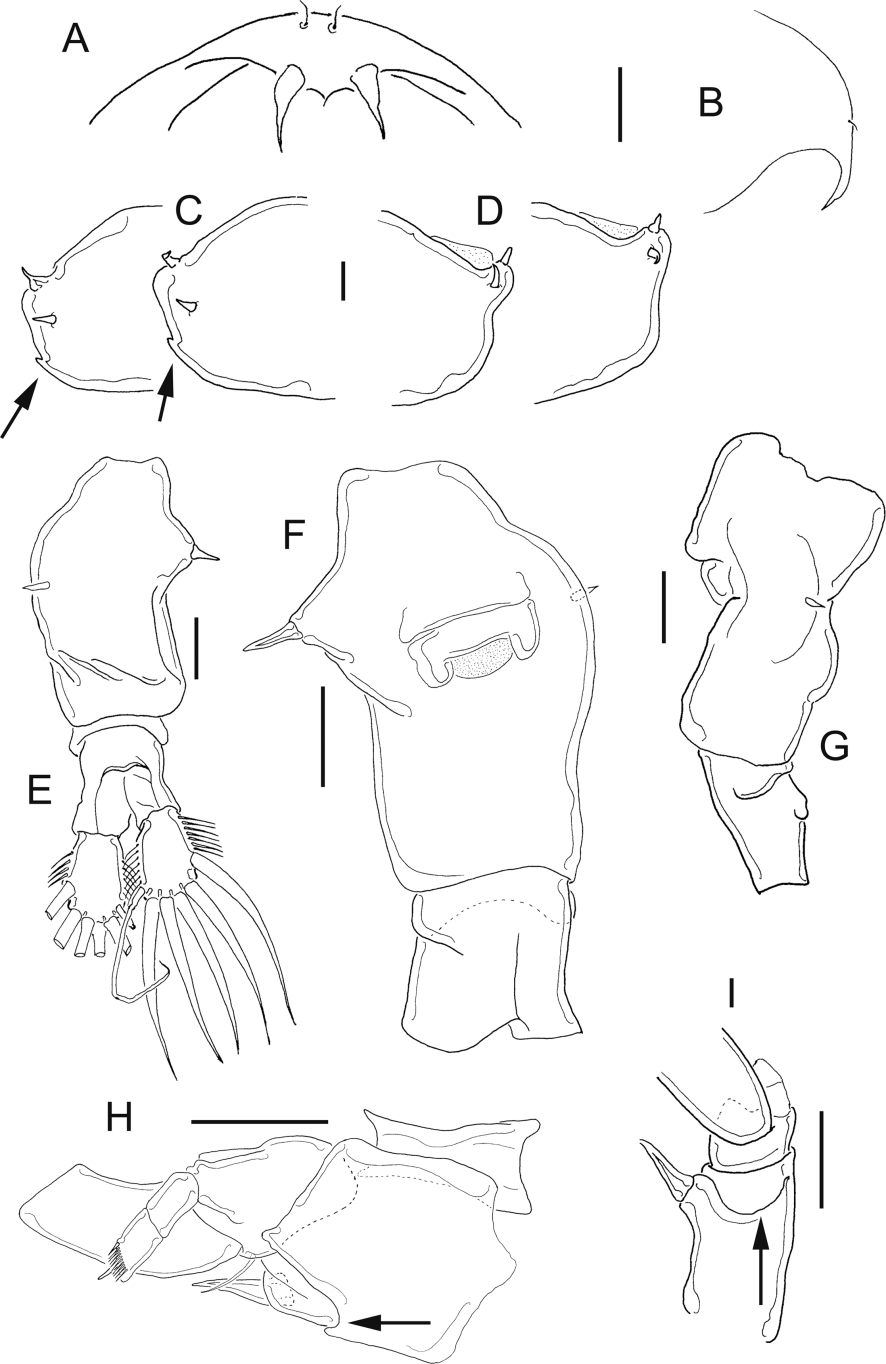
*Mastigodiaptomus cihuatlan* sp. n., adult female, holotype. (A) Rostrum, ventral. (B) Rostrum, lateral. (C) Right wing of last prosomite (latero-ventral hump arrowed). (D) Left wing of last prosomite. (E) Urosome, dorsal. (F) Genital somite, pre-anal, and anal urosomites, ventral. (G) Genital somite, pre-anal, and anal urosomites, lateral (left). (H) Fifth leg, frontal (Exp2, Exp3 omitted; antero-medial fold arrowed). (I) Fifth leg, coxa and basis, lateral, same fold arrowed. Scale bars = 50 µm.

**Figure 13:**
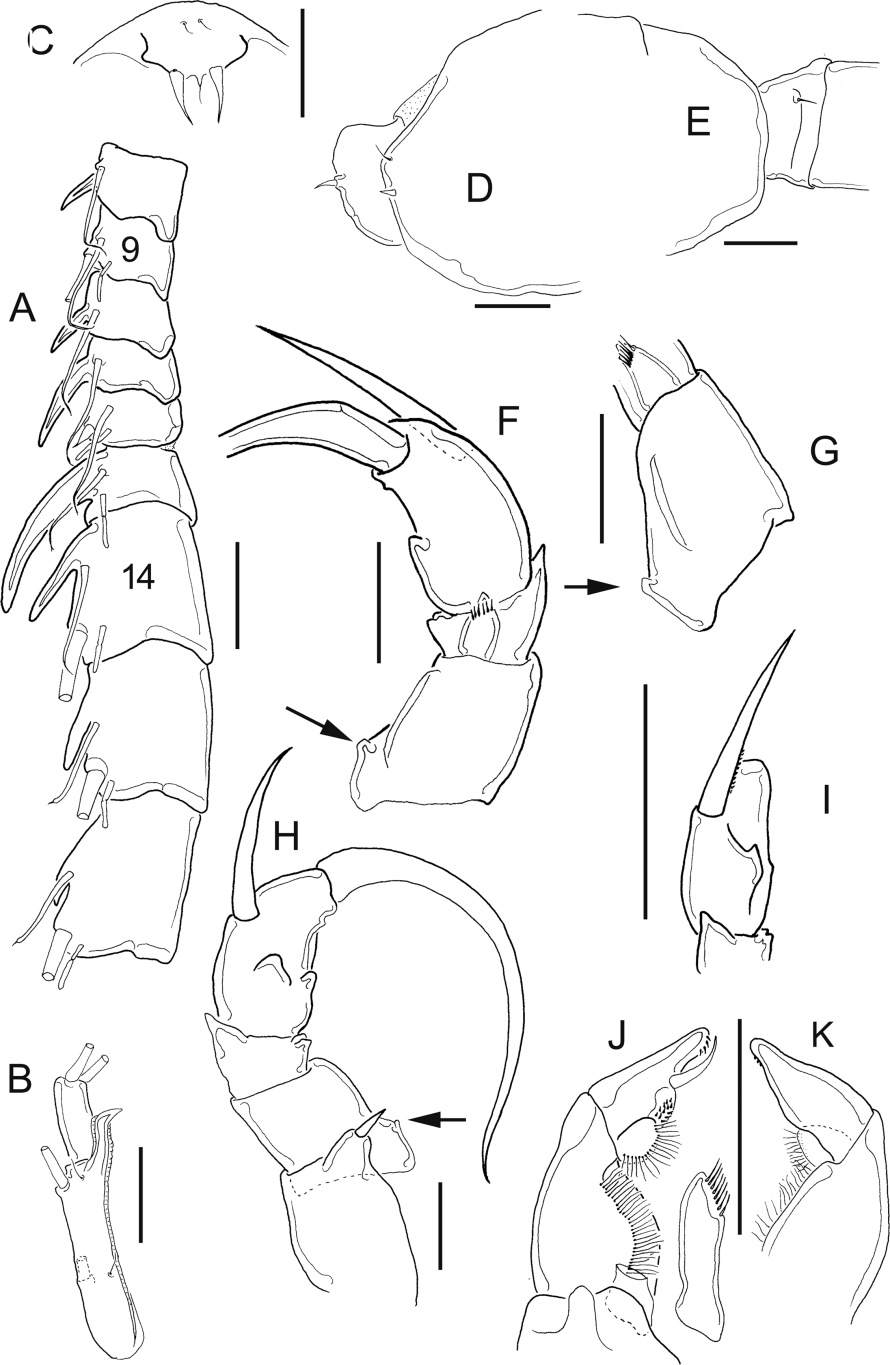
*Mastigodiaptomus cihuatlan* sp. n., adult male, allotype. (A) Right antennule, segments 8–16. (B) Right antennule, segments 20, 21. (C) Rostrum. (D) Right wing of last prosomite. (E) Left wing of last prosomite. (F) Right fifth leg, medial (angled process arrowed). (G) Fifth leg, detail of right Bsp and right Enp, medial. (H) Right fifth leg, lateral. (I) Fifth leg, detail of Exp2, lateral. (J) Left fifth leg, detail of Exp1 and Exp2 (Enp separated), frontal. (K) Left fifth leg, detail of Exp1 and Exp2, caudal. Scale bars = 50 µm.

**Holotype:** One adult female dissected on three semi-permanent slides: prosome, P1–P4 (slide 1); urosome (slide 2); P5 (slide 3) (ECOCH-Z-10322).

**Allotype:** One adult male dissected on one semi-permanent slide (ECOCH-Z-10323).

**Paratypes:** One adult male dissected on three semi-permanent slides: prosome and urosome (slide 1); A1, A2, P1–P4 (slide 2); P5 (slide 3) (ECOCH-Z-10324). Fifteen adult females and 11 adult males preserved in 90% ethanol and one drop of glycerine (ECOCH-Z-10325).

**Type locality:** Las Lagunas, Guerrero, México. Collected 29.IX.2004 (17° 52′39.1″ N; 101° 44′59.8″ W).

**Etymology:** The species name is a noun in apposition, and the term ‘cihuatlan’ means ‘women’s place’ in the Nahuatl language, which is an original language in Guerrero state. The name is dedicated to the sisters of MAGA.

**Additional material:** Two adult females and five adult males for genetic analysis from the type locality.

**Diagnosis:** Widest region of the female body on the second and third prosomites; long antennules: 6–7 antennular segments beyond the caudal rami. Antenna with 2-segmented Enp, Enp2 with two lobes: medial lobe with 7 setae, terminal lobe with 7 setae. Praecoxal arthrite of maxillule with 10 acute setae on frontal group (+4 setae on caudal group). Two praecoxal lobes on maxilla; first lobe with 5 setae, second lobe with 3 setae. Three-segmented maxillar Enp, with 1, 2 and 3 setae respectively. Genital somite asymmetric, right margin strongly acute and left margin rounded. Second urosomite partially segmented: segmentation between the second and anal urosomites, only evident dorsally (Fig. 11A). Female fifth leg strongly armed with a long spine on both coxae; this spine is almost as long as the medial margin of the basipodite (Fig. 11C). Fifth-leg coxae with a deep fold extending from the medial margin to the frontal surface (Figs. 12H and 12I).

Widest region of the male body at the level of the second and third prosomites. Antenna, maxillule, and maxilla as described for females. Asymmetric urosomite: the anal somite and both left and right rami twisted to the right (Figs. 11B and 11D). Medial margin of caudal rami hairy, but the right caudal ramus with gaps between the hairs (Figs. 11D and 11E). Dorsal surfaces from second to fourth urosomite with tiny spines (Fig. 11D). Right male antennule 22-segmented, with a strongly developed spiniform process on segments 8, 10, 11 and 13–16; antepenultimate antennular segment with one angled projection as long as the middle length of the penultimate segment (Fig. 11B). Male right fifth leg with one angulate projection on a basal bulbous widening on the right basipodite, plus a long hyaline membrane along the medial margin of the same segment. Right Exp2 with two rounded projections, one aculeus slightly longer than the bearing segment, and one long, acute apical spine (Fig. 11F).

**Female:** 1.3–1.4 mm body length, including the caudal rami. Prosomites and urosomites with smooth surfaces; last prosomite without a dorsal process. Antennule 25-segmented, last 6–7 antennular segments beyond the caudal rami (Fig. 11A). Left prosomal wing slightly more projected (distally) than the right wing (Figs. 11A, 12C and 12D). Urosome 3-segmented dorsally, plus caudal rami (Fig. 12E). Medial and lateral margins of caudal rami hairy (Figs. 11A and 12E). Long, acute rostral spines (Figs. 12A and 12B).

Antennule 25-segmented, armed as follows: (1) 1ms+1ae; (2) 3ms+1ae; (3) 1ms+1ae; (4) 1ms; (5) 1ms+1ae; (6) 1ms; (7) 1ms+1ae; (8) 1ms+1sp; (9) 2ms+1ae; (10) 1ms; (11) 2ms; (12) 1ms+1ae+1sp; (13) 1ms; (14) 1ms+1ae; (15) 1ms; (16) 1s+1ae; (17) 1ms; (18) 1s; (19) 1ms+1ae; (20) 1ms; (21) 1s; (22) 1ms+1s; (23) 1ms+1s; (24) 1ms+1s; (25) 3s+1ms+1ae.

Right wing with a low, triangular, latero-ventral hump (arrows, Fig. 12C); one large dorsal spine (which is straight or curved); and one large spine displaced towards the inner middle region of the right wing (Fig. 12C). Last prosomite bulbous, with two large spines on left side (Fig. 12D).

Genital somite asymmetric: right margin acute, bearing one large, apical spine; left margin rounded, with a short spine placed more dorsally. Genital somite slightly twisted towards the postero-dorsal region. Hair-like setae along the lateral and medial margins of the caudal rami, the lateral setae slightly thicker than the medial setae (Fig. 12E). Urosome 2-segmented ventrally (Fig. 12F): separation between the second and anal somites only evident lateral and dorsally (Fig. 12G).

**Fifth leg:** Quadrangular coxa, one large coxal spine as long as the middle length of the lateral margin of the bearing segment (Figs. 11C, 12H and 12I). From the base of the coxal spine to the frontal coxal surface, a long fold runs along the lateral coxal margin (arrow, Figs. 12H and 12I). Fifth leg with 2-segmented Enp, which bears 2 apical, long setae and short setulae diagonally arranged. On average, EnpP5 is 1.1–1.2 times longer than the medial margin of Exp1P5 (Fig. 12H). Exp2 with a row of strong spinules along both margins; one lateral, short spine placed next to Exp3. The latter is separated and bears one long plus one short spine (Fig. 11C).

**Male:** 0.97–1.2 mm body length, including the caudal rami. Rostral spines long, acute (Fig. 13C). Cuticular surface of prosomites smooth; tiny spinules on dorsal surfaces of urosomites (Fig. 11D). First urosomite with a large spine on the right lateral margin (Fig. 13D) and one tiny hair-like setule on the left margin (Fig. 13E). Anal somite and caudal rami twisted to the right side; right caudal ramus with angular polygonal thickenings on dorsal and ventral surfaces, arranged as keels; medial margin of right ramus less pilose than medial margin of left caudal ramus (Figs. 11D and 11E).

Right geniculate antennule 22-segmented, spine on segment 8 modified into a long spiniform process that reaches a third of the length of segment 9; also a spiniform process on segments 10, 11 and 13–16 (Fig. 13A). Spiniform process on segment 10 reaching the half length of the next segment. Spiniform process on segment 11 reaching the distal margin of segment 12 (Fig. 13A).

Right A1 ornamented as follows: (1) 1ms+1ae; (2) 3ms+1ae; (3) 1ms+1ae; (4) 1ms; (5) 1ms+1ae; (6) 1ms; (7) 1ms+1ae; (8) 1ms+1sps; (9) 2ms+1ae; (10) 1ms+1sps, (11) 1ms+1sps; (12) 1ms+1ae+1sp; (13) 1ms+1ae+1sps; (14) 2ms+1ae+1sps; (15) 2ms+1ae+1sps; (16) 2ms+1ae+1sps; (17) 1ms; (18) 0; (19) 1ms+1ae; (20) 1ms+1s+2ae; (21) 2s; (22) 4s (Fig. 13A). Antepenultimate antennular segment with a hook-like projection strongly angled distally, that reaches the half length of the penultimate antennular segment; with a corrugated lateral hyaline membrane that extends along the entire segment length (Fig. 13B). Left male antennule similar to the female.

Right wing of the last prosomite with one thin dorsal seta and one large ventral spine (Fig. 13D); left wing marginally smooth (Fig. 13E).

Right fifth leg, caudal view: Coxa with one lateral spine (Fig. 11F). Basipodite bulbous and with one angled basal process (process arrowed in Figs. 13F–13H), plus a long hyaline membrane that extends along the medial margin of the segment (Figs. 11F, 13F and 13G). Right Exp1 with a triangular, lateral apex and two rounded lamellae on the medial margin (Fig. 11F). Right Enp1 slightly longer than the medial margin of the right Exp1 (Figs. 13F and 13G). Lateral margin of Exp2 curved; surface of the right Exp2 with two sclerotised (basal) projections (Figs. 11F, 13F, 13H and 13I) and one sclerotised line that forms a small triangular hump distally (Figs. 13F and 13H). One straight aculeus inserted in half of Exp2 and longer than the bearing segment, plus one long acute apical spine, which is as long as the entire right P5 (Fig. 13H).

Left fifth leg: Quadrangular coxa with a large lateral spine; basipodite with lateral seta, rectangular (Fig. 11F). Medial margin of Exp1 pilose, as long as the left Enp (Fig. 13J). Exp2 triangular, apex with tiny spinules plus a curved seta in frontal view; basal region widened, with a rounded, pilose lobe (Figs. 13J and 13K).

**Distribution:** Mexico, recorded only in Las Lagunas, Guerrero state.

*Mastigodiaptomus patzcuarensis* (Kiefer, 1938)

Figures 14–16

**Figure 14:**
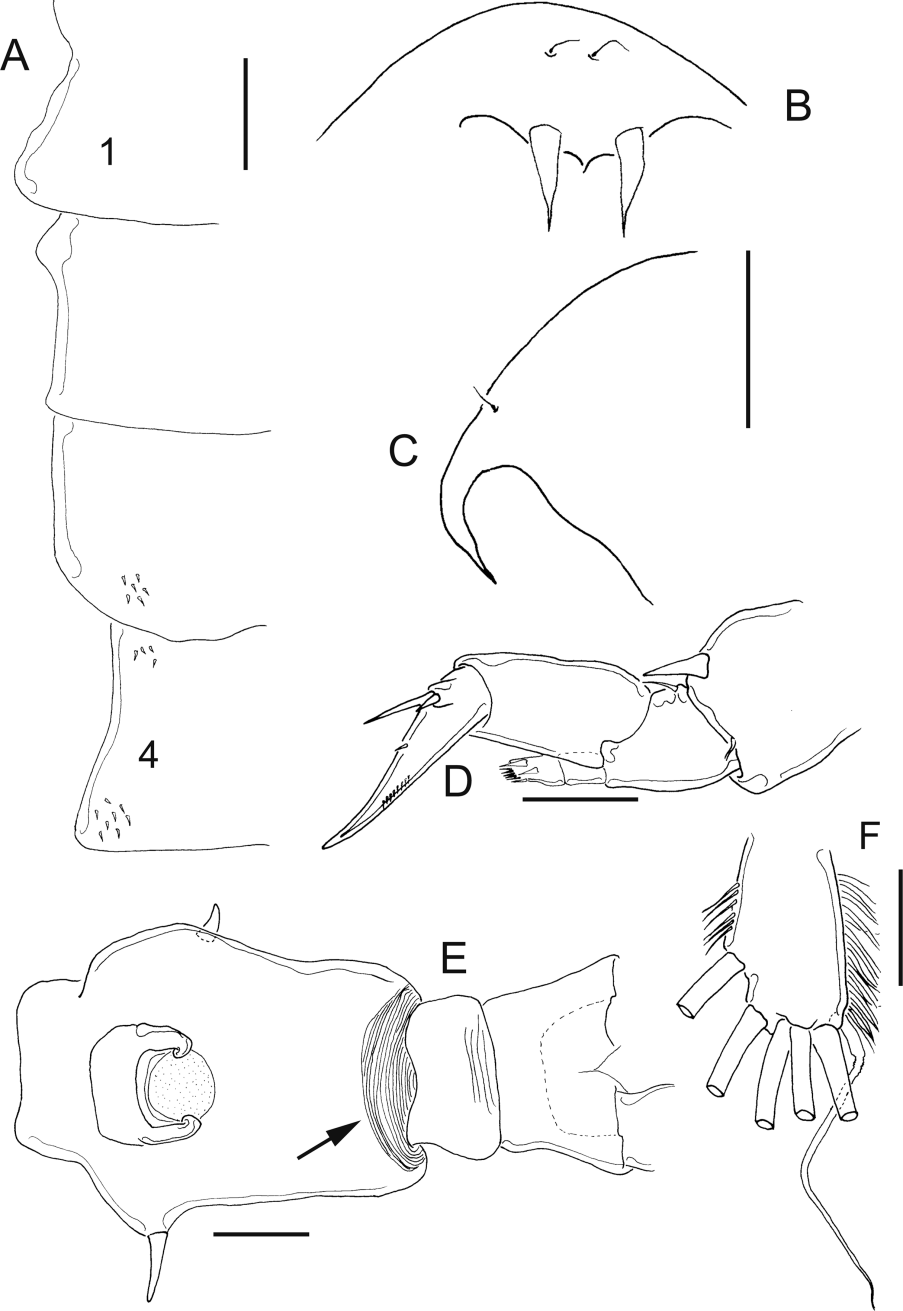
*Mastigodiaptomus patzcuarensis* (Kiefer, 1938), adult female. (A) Prosomites, ventro-lateral. (B) Rostrum, ventral. (C) Rostrum, lateral. (D) Fifth leg, caudal. (E) Genital somite, pre-anal, and anal urosomites, ventral; detail of cuticular sclerotizations arrowed. (F) Caudal ramus, ventral. Scale bars = 50 µm.

**Figure 15:**
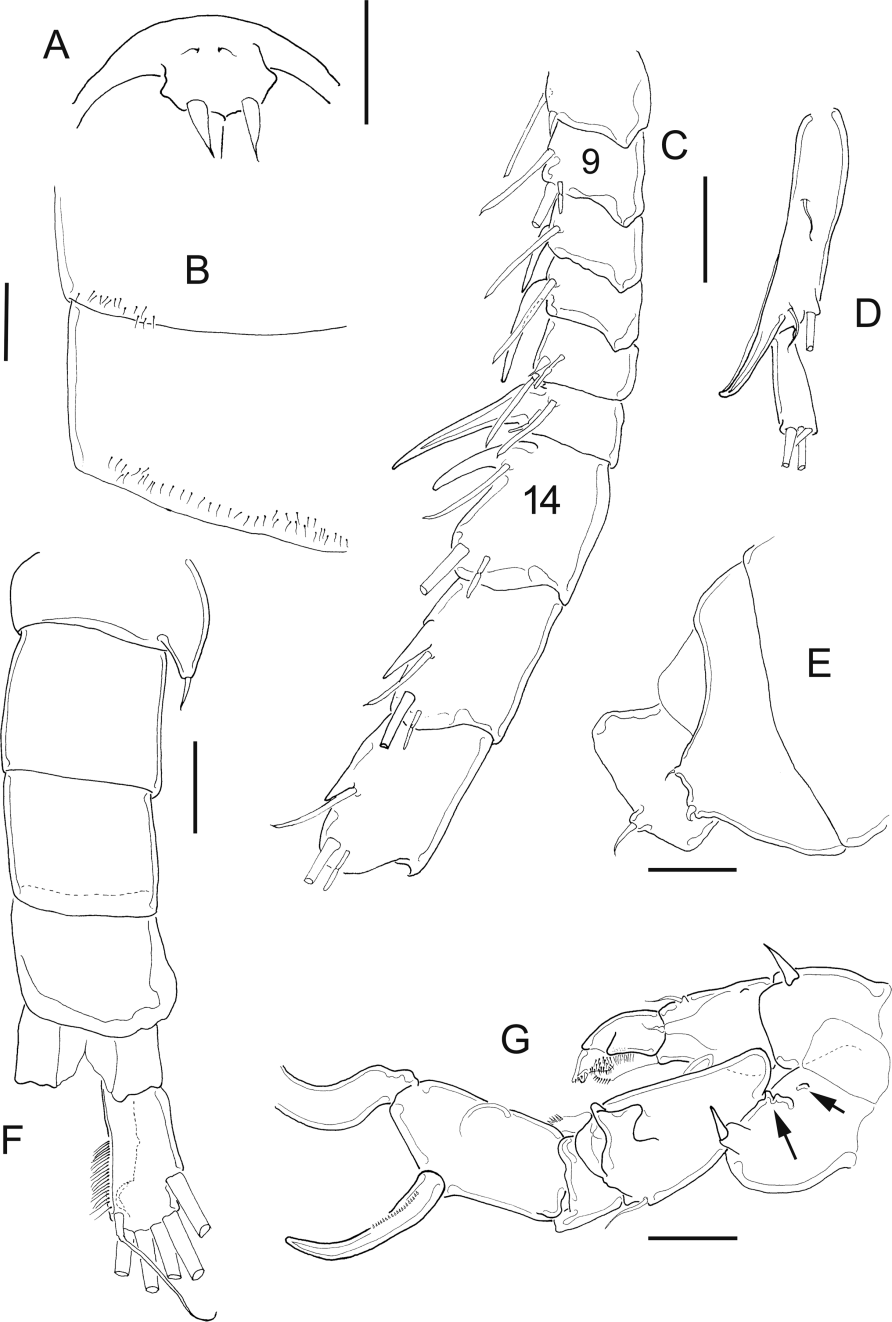
*Mastigodiaptomus patzcuarensis* (Kiefer, 1938), adult male. (A) Rostrum. (B) Prosomites 3 and 4, lateral. (C) Right antennule, segments 8–16. (D) Right antennule, segments 20, 21. (E) Right wing of last prosomite. (F) Urosomites, dorsal (left furcal ramus omitted). (G) Fifth leg, caudal; lobes and protrusion arrowed. Scale bars = 50 µm.

**Figure 16:**
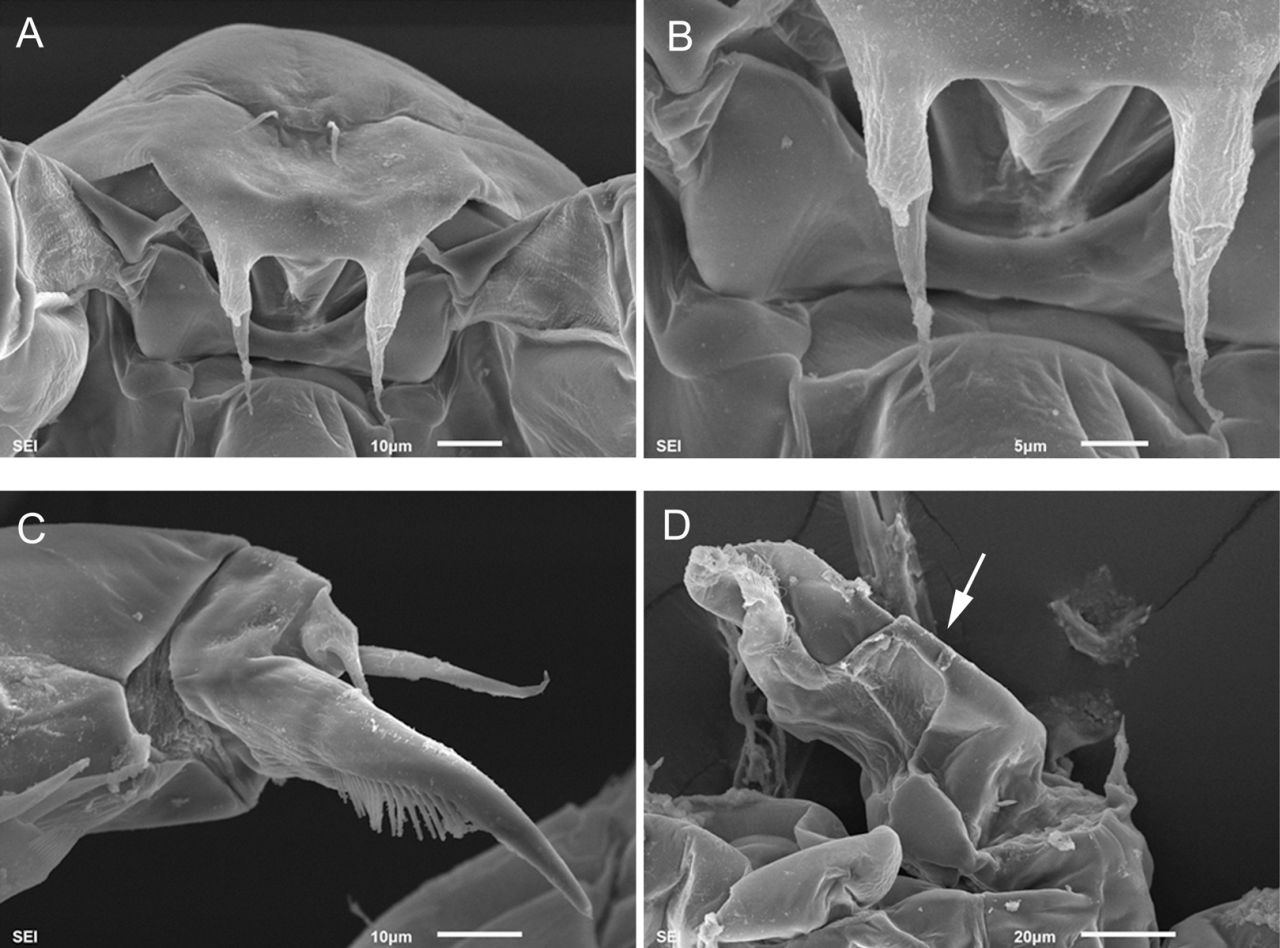
*Mastigodiaptomus patzcuarensis* (Kiefer, 1938), adult female (A–C) and adult male (D). (A) Rostrum. (B) Detail of rostrum. (C) Fifth leg, detail of Exp2 and Exp3. (D) Fifth leg, detail of Bsp, caudo-lateral.

*Diaptomus albuquerquensis patzcuarensis* Kiefer, 1938

*Mastigodiaptomus patzcuarensis* Gutiérrez-Aguirre, Cervantes-Martínez & Elías-Gutiérrez, 2014: 7–8; figs. 7, 8.

**Material:** Eighteen adult females and males for genetic analysis; five adult females and five adult males for SEM analysis, and 30 adult females and 30 adult males. All these material from Lago Chapultepec, Ciudad de México; collected 25.XII.2018 (ECOCH-Z-10328) (19° 25′ 24.6″ N; 99° 11′ 09.42″ W).

**Female:** 1.1–1.2 mm body length, including the caudal rami; the last two antennular segments are beyond the distal margin of the caudal rami. Last prosomite with a dorsal process. Latero-ventral region of the third and fourth prosomites with a few, small spines (Fig. 14A). Rostrum with spines 3.5–3.6 times longer than wide, distally attenuated (Figs. 14B, 14C; 16A and 16B).

Antennule 25-segmented, armed as follows: (1) 1ms+1ae; (2) 3ms+1ae; (3) 1ms+1ae; (4) 1ms; (5) 1ms+1ae; (6) 1ms; (7) 1ms+1ae; (8) 1ms+1sp; (9) 2ms+1ae; (10) 1ms; (11) 2ms; (12) 1ms+1ae+1sp; (13) 1ms; (14) 1ms+1ae; (15) 1ms; (16) 1s+1ae; (17) 1ms; (18) 1s; (19) 1ms+1ae; (20) 1ms; (21) 1s; (22) 1ms+1s; (23) 1ms+1s; (24) 2s; (25) 3s+1ae+1ms.

Fifth leg Exp1 is 1.9–2.0 times longer than Enp (Fig. 14D); the latter is 2-segmented, with 2 long apical setae and short setules diagonally arranged (Figs. 14D and 16C). Two spines on Exp3P5, and Exp2 with a row of spines medially (Fig. 16C).

Last prosomite projected on left side, with two large spines. Genital double somite is 1.3–1.4 times longer than wide, clearly asymmetrical: right margin laterally acute and with a strong spine inserted more proximally than the left spine, which is on a sinuous margin (Fig. 14E). Left spine of genital double somite inserted at 45% of the segment length. Some parallel, curved cuticular sclerotizations near posterior margin of the genital double somite and pre-anal somite (arrow, Fig. 14E). Caudal rami with hair-like setae along the medial margin and spine-like setules along the lateral margins (Fig. 14F).

**Male:** 0.9–1.0 mm body length, including the caudal rami. Rostrum with spines 3–4 times longer than wide (Fig. 15A). Posterior margin of third and fourth prosomites with a row of hair-like setules (Fig. 15B); the rest of prosomites are smooth. Right male antennule 22-segmented, with a large spiniform process on segments 10, 11 and 13–16. The tip of the spine on segment 10 reaches the half length of segment 11. The tip of the spine on segment 11 is beyond the distal margin of the next antennular segment (Fig. 15C).

Segments of right geniculate antennule armed as follows: (1) 1ms+1ae; (2) 3ms+1ae; (3) 1ms+1ae; (4) 1ms; (5) 1ms+1ae; (6) 1ms; (7) 1ms+1ae; (8) 1ms+1sp; (9) 2ms+1ae; (10) 1ms+1sps, (11) 1ms+1sps; (12) 1ms+1ae+1sp; (13) 1ms+1ae+1sps; (14) 2ms+1ae+1sps; (15) 2ms+1ae+1sps; (16) 2ms+1ae+1sps; (17) 1ms; (18) 1ms; (19) 1ms+1ae; (20) 1ms+1s+2ae; (21) 2s; (22) 4s (Fig. 15C). Antepenultimate antennular segment is 3.7–3.9 times longer than wide, with a hook-like projection that bears a smooth hyaline membrane (Fig. 15D).

Left wing of last prosomite with one large spine ventrally and one thin seta dorsally; first urosomite with one short setule placed laterally on the left margin. Right wing with two spines, the ventral spine larger than the dorsal spine; first urosomite with a large spine placed distally on the right margin (Figs. 15E and 15F). Ventral and dorsal surfaces of all urosomites smooth; distal margin of the fourth urosomite slightly projected on right side. Caudal rami pilose medially; right ramus with a bulbous hump on the ventral surface (Fig. 15F).

Right fifth leg, caudal: The intercoxal sclerite, and the coxa folded towards the left but not strongly projected. Coxa with one lateral spine, one tiny bi-lobed fold, and one tiny rounded protrusion (arrowed in Fig. 15G). Basipodite basally protruded, with lateral seta and with two hyaline membranes in caudal view: one rounded, medial; and one angled, distal. Enp slightly longer than Exp1; the latter with rounded protuberances on the medial and lateral margins. Right Exp2 with one curved, medial hyaline membrane and one basal angulate thickening. In the distal third, a curved aculeus is inserted, which is shorter than the bearing segment: ratio between the length of Exp2 and the length of the aculeus = 1.2 (Fig. 15G). Curved distal spine is 2.4–2.5 times longer than Exp2.

Left fifth leg: Coxa with one large, lateral spine (Fig. 15G). Basipodite with one lateral seta, one tiny, rounded projection in the basal region, plus an inverted Y-shaped wrinkle (arrowed in Figs. 15G and 16D), which produces a cuticular thickening in the next segment, visible with light microscopy. Endopod and Exp1 with similar lengths, the latter medially pilose. Exp2 triangular, distally angulate, medially pilose, with a delicate distal seta and 2 or 3 tiny spinules (Fig. 15G).

**Distribution:** Restricted to Mexican highlands (Central Plateau), Chapultepec Lake, Mexico City; Ignacio Ramírez and La Goleta reservoirs, Estado de México state (19.461 N, 99.797 W); Atoyac River and Flor del Bosque reservoir, Puebla state (19.036 N, 98.228 W); Atlangatepec Lake, Tlaxcala state (19.56 N, 98.196 W); Pátzcuaro Lake, Michoacán state (type locality 19.597 N, 101.652 W); and La Cruz, Guanajuato state (21.193 N, 100.574 W).

### Genetic analysis

**Figure 17:**
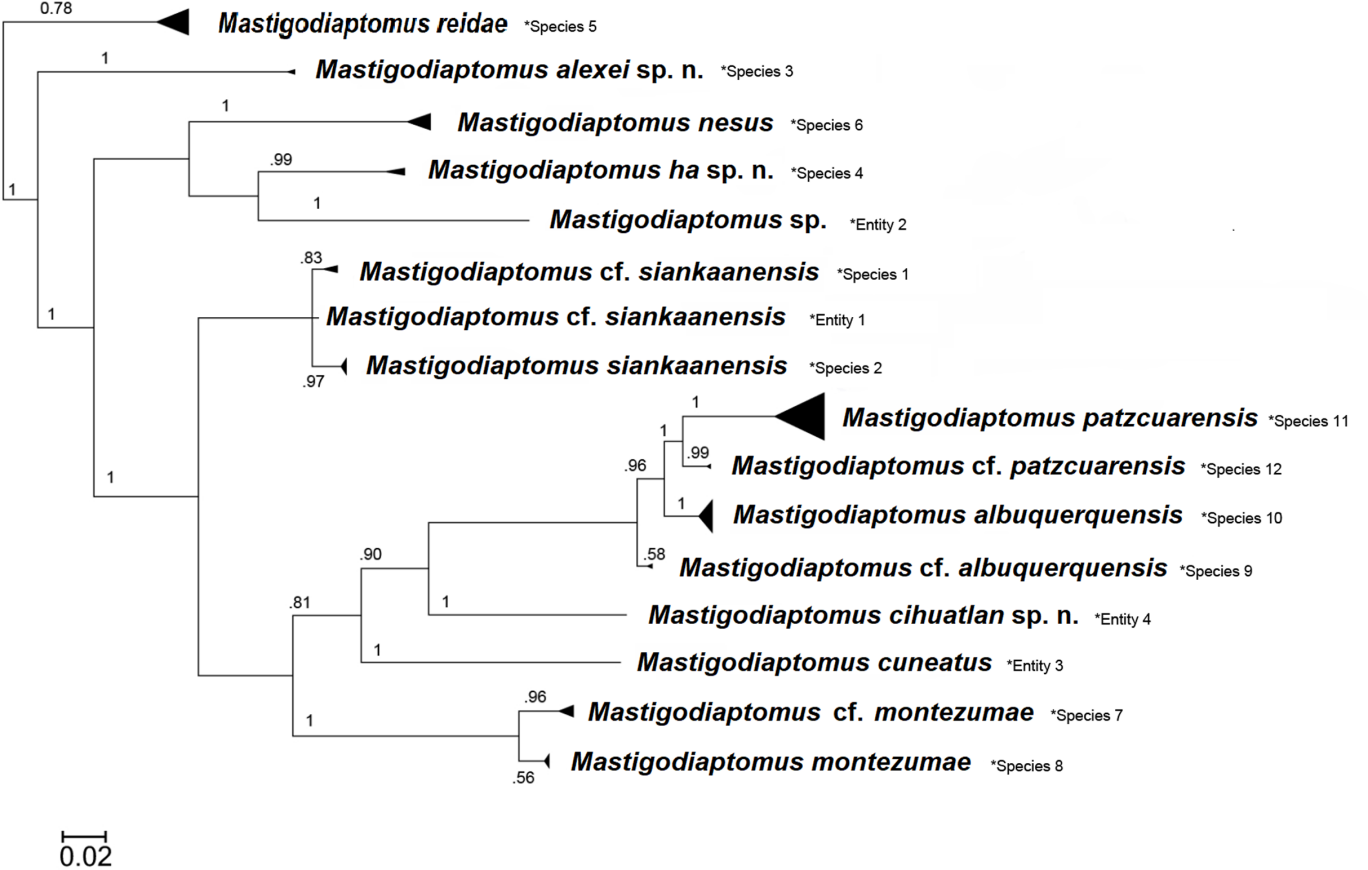
Bayesian tree of COI sequences for 203 specimens of *Mastigodiaptomus* (16 clades). Branches are collapsed for each recognised clade; values on each branch are the posterior probabilities. Species delimited by the general mixed yule coalescent model (GMYC) are after the Linnaean name in each clade.

DNA analyses revealed 16 clades within the genus (Fig. 17) based on a total of 203 sequences that were collapsed into 86 different haplotypes by ALTER software. All records were downloaded from the BOLD database and verified for correct identification. After alignment, we obtained a maximum length of 571 bp, and one sequence was 468 bp in length. With the exception of this sequence, all the sequences were over 500 bp in length. The average distance within species of the genus was 2.31% and the maximum 12.19%, this latter was observed in *M. montezumae* (Brehm, 1955), which requires further study for clarification. *Mastigodiaptomus siankaanensis* Mercado-Salas, Khodani, Kihara, Elías-Gutiérrez and Martínez-Arbizu, 2018, was splitted in two clades and one entity, due to the addition of more sequences than a previous study (Mercado-Salas et al., 2018). They also require a more detailed analyses of south and north populations in Yucatan Peninsula.

Divergences within species of the five taxa analysed here are included in Table 2. In all species, values were under the 3.0% threshold, except in *M. patzcuarensis*, the only species with a wide distribution in the Mexican Plateau with two BIN assignments. In the case of *M. patzcuarensis*, the maximum was 4.78%, and it was possible to distinguish two sub-clades, but there was no biogeographical justification for separate them because the populations seemed to be intermixed, possibly due to passive dispersal of resistant eggs.

**Table 2:** Pairwise COI sequence divergence within the *Mastigodiaptomus* species analysed. Percentage of maximum and minimum K2P intraspecific distances of the species studied here. The Barcode Index Number (BIN) assigned by the BOLD, GC composition and localities where the species were collected are also presented.

Species	N	Comparisons	Min dist (%)	Mean dist (%)	Max dist (%)	SE dist (%)	BIN assigned	GC composition (mean ± SE)	Locality
*M. alexei* sp. n.	3	3	0.31	0.62	0.92	0.08	AAF1220	41.31 ± 0.2	Camalote
*M. ha* sp. n.	5	10	0.00	0.08	0.23	1.01	AAU1038	38.76 ± 0.2	Siete Bocas Verde Lucero
*M. cihuatlan* sp. n.	1	NA	NA	NA	NA	NA	AAJ1590	40.45	Las Lagunas
*M. nesus*	9	36	0.00	1.10	2.57	0.02	AAG9522	45.51 ± 0.3	Minicenote El Padre La Esperanza
*M. patzcuarensis*	41	472	0.00	1.53	4.78	0.00	AAC4008 ABZ7048	41.31 ± 0.17	Pátzcuaro Chapultepec Flor del Bosque Cuitzeo La Goleta Ignacio Ramírez La Cruz

The GC content ranged from 38.76 ± 0.2% to 45.51 ± 0.3% in the five studied species (Table 2), and all values were near the range for all species of the genus (42.77 ± 0.14).

The number of species suggested by the GMYC analyses with a confidence interval of 11–19 gave 12 species plus 4 entities (species inferred from one sequence) consistent with the clades from the Bayesian analyses. The likelihood of GMYC model (539.65) was significantly superior to the null model (535.23) (see Fig. 17).

## Discussion

The first diaptomid species described in Mexico as a new species belongs to the genus *Mastigodiaptomus*; it was called *M. lehmeri* Pearse, 1904. Later, this species was synonymised with *M. albuquerquensis* by Marsh (1907). Suárez-Morales & Elías-Gutiérrez (2003) described this process in detail.

In Mexico, *Mastigodiaptomus* has been recorded in water systems from between 1,600 and 2,400 m.a.s.l. in the Neovolcanic Axis Province (Dos Santos-Silva, Elías-Gutiérrez & Silva-Briano, 1996) to other regions, such as semi-desertic provinces, including the Mexican Altiplano (Gutiérrez-Aguirre, Cervantes-Martínez & Elías-Gutiérrez, 2014). The genus is also found in surface waters in the Caribbean Province at lowlands (Bowman, 1986; Mercado-Salas et al., 2018).

This Neotropical genus is clearly able to inhabit a wide range of habitats. Therefore, *Mastigodiaptomus* is the diaptomid genus with the highest diversity in Mexico: the number of currently known species (including the ones described in this paper) is higher than the number of *Leptodiaptomus* species (13 vs. 7, respectively) and is comparable to the number of species of two other freshwater, free-living cyclopoid genera also distributed in Mexico. These are *Mesocyclops*, with 12 species (Suárez-Morales & Gutiérrez-Aguirre, 2001), and *Eucyclops*, with 17 (Mercado-Salas, Suárez-Morales & Silva-Briano, 2015).

The diagnostic features of the *Mastigodiaptomus* genus (Kiefer, 1932; Wilson & Yeatman, 1959; Dussart & Defaye, 1995) are: segment 11 of the antennules of females and left antennule of males with two setae; coxa of female P5 with a long, spatulated seta; one lateral spine on Exp1P1 in females and males; Enp of right male P5 longer than the right Exp1; right male antennule with a spiniform process on segments 10, 11 and 13–16; and basis of the right male P5 with hyaline lamellae on the caudal side.

Morphological features such as the presence of one lateral spine on Exp1 of P2–P4, the fused Exp2–Exp4 antennal segments, the presence of one seta on antennal Exp1, the Exp2P5 as one unarticulated spine-like process in females, or the right geniculate A1 and P5 in males have supported the monophyletic clade of Diaptomidae and related families in earlier phylogenetic studies by Park (1986) and Bradford-Grieve et al. (2010). Recently, a multi-gene phylogeny, that includes analysis of COI, is consistent with that monophyletic relationships at Family and Superfamily level (Blanco-Bercial, Bradford-Grieve & Bucklin, 2011) and according with the performed analysis, all these features are present in the species of *Mastigodiaptomus*.

*Mastigodiaptomus alexei* sp. n. and *M. ha* sp. n., are similar to *M. texensis* described by Wilson (1953), but several features can be used to differentiate the species. The following features can be useful to differentiate females: in *M. texensis*, the antennule is longer than that in *M. alexei* sp. n. and *M. ha* sp. n; when the length/width ratios of the genital double somite are compared, that of *M. texensis* is the shortest, followed by that of *M. ha* sp. n., and that of *M. alexei* sp. n., is the longest. The symmetries of the genital somite are different: in *M. texensis*, it is symmetric, and the positions of the left and right spines are at the same level in the segment, while in *M. ha* sp. n. and *M. alexei* sp. n., the right spine is positioned more proximally than the left spine.

In males, features of the right antennules and fifth legs can differentiate species: the length of the spinal processes on segment 10 and 11 of the right A1 is shorter in *M. texensis* than in *M. alexei* sp. n. and *M. ha* sp. n. The position of the spinal process on segment 16 is more distal in *M. texensis* than in *M. alexei* sp. n. and *M. ha* sp. n. The length ratio between left Exp1P5 and left Exp2P5 is highest in *M. texensis*, intermediate in *M. ha* sp. n., and lowest in *M. alexei* sp. n. The caudal surface of the right Exp2P5 is smooth in *M. ha* sp. n. but with one crescent-shaped sclerotisation in *M. texensis* and *M. alexei* sp. n. The ornamentation of the male right basis of P5 is different among species (see Supplemental File 3). Therefore, a combination of at least 20 morphological features was observed to differentiate among the species analysed.

Some morphological features of *M. cihuatlan* sp. n. are unique in the genus and can be useful in classifying the species: in females, the asymmetric genital double somite is twisted; the ventral spine is dorsally displaced on the fifth right wing; the anal and pre-anal somites are fused ventrally, and a large fold extends from the caudal, medial margin to the frontal surface of the fifth leg coxopodite. In males, unique features that are useful for distinguishing *M. cihuatlan* sp. n. from other *Mastigodiaptomus* species include a spiny, asymmetric, twisted urosomite; the spine of the eight antennular segment modified into a strong spiniform process; and the complex ornamentation of the right basis of the fifth leg.

Detailed morphological analysis performed here revealed that *M. nesus* is a species with extremely small ornaments on the prosoma and urosoma of adult females and males (see Figs. 1 and 2). This feature was not noted in the original description of *M. nesus* by Bowman (1986) but was detected in the populations of San Salvador Island (type locality) and was a stable character in all the populations from Quintana Roo surveyed here.

Cuticular surface ornamentation of the body is also observed in the females of *M. albuquerquensis* (Herrick, 1895), females and males of *M. patzcuarensis* (Gutiérrez-Aguirre, Cervantes-Martínez & Elías-Gutiérrez, 2014) and males of *M. suarezmoralesi* (Gutiérrez-Aguirre & Cervantes-Martínez, 2013) whereas *M. siankaanensis* bears hair-like setulae only on one cephalic appendage, the female antennulae (Mercado-Salas et al., 2018). In the Americas, only two species belonging to *Leptodiaptomus* have been recorded as having cuticular ornamentation on the dorsal surfaces of urosomites. They are *L. siciloides* (Lilljeborg, 1889) and *L. connexus* (Light, 1938), which are distributed from the western and southwestern USA to the northern region of Mexico. We consider that this kind of ornamentation should be considered in further descriptions.

The number of elements on each segment of A1 is stable specially in females when different species of *Mastigodiaptomus* are compared. However one modified seta replaces one seta, in the antennular segments 18, 21 and 24 in *M. alexei* sp. n. or in the segment 24 in *M. cihuatlan* sp. n.

In males more differences were observed (in the species surveyed here) when the number of elements of each antennular segment are compared. For instance, the segment 9 bears two elements in *M. alexei* sp. n., whereas in the other species, three elements were observed. *Mastigodiaptomus nesus* lacks one aesthetasc in segment 13; or in *M. nesus* and *M. patzcuarensis* the segment 18 bears one modified seta; but in *M. ha* sp. n. there is one spine, or in *M. alexei* sp. n. and *M. cihuatlan* sp. n., the segment 18 has not elements. Then, this kind of ornamentation also seems informative to define morphologically to *Mastigodiaptomus* species.

The results for these new species confirm the results of previous analyses of the genus (Mercado-Salas et al., 2018), who analysed 11 species, with one exception. The GMYC analysis suggests that the two haplotypes of *M. siankaanensis* found in her work could represent two different species, plus a third one represented by a singleton. Also, further analyses of *M*. cf. *montezumae* and *M*. cf. *patzcuarensis* are needed (mainly of populations from semi-desert pools).

Although the three new species are represented by few sequences (1–5), they are well supported, not only by the DNA barcodes. The conclusion based on morphological diagnostic characters is consistent with that based on GMYC analyses and the BIN system of the BOLD database (see Supplemental File 2) for these three species (Ratnasingham & Hebert, 2013).

According to the K2P analysis, the minimum and maximum interspecific distances between COI sequences within *Mastigodiaptomus* are 5.21 and 26.48%, respectively, which are consistent with the findings of Mercado-Salas et al. (2018). As shown in Fig. 17, *M. patzcuarensis* is a well-defined clade, but among all the sequenced species of *Mastigodiaptomus*, this is the species with the highest intraspecific genetic divergence; the population (females and males) from Chapultepec Lake exhibited almost all of the morphological features previously described as typical for *M. patzcuarensis* (Gutiérrez-Aguirre, Cervantes-Martínez & Elías-Gutiérrez, 2014); however, one morphological feature was notably variable between populations. The presence of rows of hair-like setae on the prosomites is continuous in the Pátzcuaro, Flor del Bosque, and Ignacio Ramírez populations, but interrupted in the Chapultepec population and lacking in La Goleta, La Cruz and Cuitzeo populations. Additionally, the population from Chapultepec has extremely small rounded projections on the right coxa and left basis of the male fifth leg (arrows in Fig. 15G), which were not observed in the rest of the mentioned populations of *M. patzcuarensis*. Further studies are necessary to establish whether these genetic and morphological variations reflect reproductive isolation.

Two others rare *Mastigodiaptomus* recently described are *M. cuneatus* Gutiérrez-Aguirre & Cervantes-Martínez, 2016, found only in a lagoon within the city of Mazatlán (Mexico) and *M. suarezmoralesi* Gutiérrez-Aguirre & Cervantes-Martínez, 2013, described from the unique Montebello lakes in Chiapas state, Mexico. The latter species still has not been studied with DNA barcodes. Other species in need of attention with a restricted distribution are *M. purpureus* Marsh, 1907, found in Cuba, and *M. amatitlanensis* M. S. Wilson, 1941, from the Amatitlan Lake (Guatemala) (Wilson & Yeatman, 1959). The latter species may be almost extinct because it has not been detected in several surveys of this lake (A. Cervantes-Martínez, 2019, personal communication).

The next goal is understanding the distribution of these new species because after extensive samplings in Mexican freshwater systems, they appear to have only a very restricted distribution. This case is not rare because *M. maya* Suárez-Morales & Elías-Gutiérrez, 2000; which may be the largest representative of the genus, with a length of more than 2.3 mm (excluding the furcal rami) (Suárez-Morales & Elías-Gutiérrez, 2000), has not been observed since its original description, even in the type locality, an ephemeral pool in a forest (M. Elías-Gutiérrez, 2019, personal communication).

Finally, the level of analysis presented here, considering the specimen number and species number analysed with the COI marker of the *Mastigodiaptomus* genus is only comparable to that of the studies on *Moina* (Cladocera: Anomopoda), another microscopic freshwater crustacean distributed in Mexico and the World (Elías-Gutiérrez et al., 2019; Montoliu-Elena, Elías-Gutiérrez & Silva-Briano, 2019). In these cladocerans, the intraspecific distances ranged from 3.72 to 18.09% and thus were lower than the intraspecific distances found for *Mastigodiaptomus*.

## Conclusions

These findings show that in *Mastigodiaptomus*, not only the morphological differences in right male antennule, or the fifth legs but also several morphological cuticular characters agree with the interspecific genetic differences higher than 3% observed in sequences of the COI gene.

Three new species of *Mastigodiaptomus* were described based on their genetic and morphological features which are *M. alexei* sp. n., *M. ha* sp. n. and *M. cihuatlan* sp. n. The analysis allowed amended descriptions of *M. nesus*, and *M. patzcuarensis* s. str.

*Mastigodiaptomus* is the diaptomid genus with the highest diversity in Mexico (13 species described), followed by *Leptodiaptomus*, with 7 known species. Although this Neotropical genus is clearly able to inhabit a wide range of freshwater habitats, several species of *Mastigodiaptomus* appear to have a restricted distribution in Mexico. If is the case, some protection measures should be provided in the water bodies where they dwell in order to avoid extinctions (possibly already happened with *M. amatitlanensis*). This kind of studies could provide the bases to protect the freshwater microfauna.

### Key to the species of *Mastigodiaptomus*

The key is based on morphological analysis similar to those generated in the Systematic section of this manuscript or on original descriptions (Wilson, 1953; Suárez-Morales & Elías-Gutiérrez, 2000; Gutiérrez-Aguirre & Cervantes-Martínez, 2013; Gutiérrez-Aguirre, Cervantes-Martínez & Elías-Gutiérrez, 2014; Mercado-Salas et al., 2018).

### Males

1 Spiniform process on segment 16 of right antennules strongly developed, almost as long as its segment width; right basis of P5 with one basal subrectangular protuberance on medioproximal margin*Mastigodiaptomus reidae* Suárez-Morales & Elías-Gutiérrez, 2000

–Spiniform process on segment 16 of right antennules reduced (Fig. 4A) or absent; right basis of P5 with basal rounded protuberance, with basal angled protuberance (Fig. 13F) or without basal protuberance (Fig. 2D) on medioproximal margin2

2 Right basis of P5 with only one lobular protuberance on medioproximal margin, no chitinous lamella or lamellae (in caudal view)3

–Right basis of P5 with chitinous lamella or lamellae (Figs. 2D, 4F and 8F) and with or without protuberance on medioproximal margin (in caudal view)4

3 Lobular protuberance of right basis of P5 large; right Exp2 of P5 with two semi-circular transverse lamellae and one longitudinal ‘Y’-shaped ridge; antepenultimate antennular segment with a fang-like process*M. montezumae* (Brehm, 1955)

–Lobular protuberance of right basis of P5 short; right Exp2 of P5 with one low, rounded protuberance on outer margin; antepenultimate antennular segment with wide, knob-like process*M. maya* Suárez-Morales & Elías-Gutiérrez, 2000

4 Right basis of P5 with one chitinous lamella (or hyaline membrane) (Fig. 2D)5

–Right basis of P5 with more than one chitinous lamella (or hyaline membrane) (Fig. 15G)12

5 Caudal surface of right basis of P5 with chitinous semicircular lamella (Figs. 4F, 6E, 8F and 10G)6

–Caudal surface of right basis of P5 with chitinous cuneiform, transversal; longitudinal (Figs. 11F and 13G) or semi triangular lamella10

6 Caudal surface of right Exp2 of P5 smooth, without lamellae (Figs. 8F and 10H) *Mastigodiaptomus ha* sp. n.

–Caudal surface of right Exp2 of P5 ornamented with sclerotisation (Fig. 4F), lamellae, hyaline membranes, or ridge (Fig. 2D)7

7 Caudal surface of right Exp2 of P5 with two short semi-circular lamellae: one medial and one lateral; aculeus length is 50% of the length of its segment; short spiniform process on antennular segment 14*Mastigodiaptomus purpureus* Marsh, 1907

–Caudal surface of right Exp2 of P5 with one sclerotisation; aculeus length more than 50% of the length of its segment (Figs. 2D and 3E); long spiniform process on antennular segment 14 (Fig. 4A)8

8 Urosomites 2–4, with rows of tiny spines on dorsal surfaces (Fig. 2C); left Exp2 triangular, attenuated distally (with only hair-like setae) (Fig. 2F); right Exp2 with a long, straight ridge (Fig. 2E)*Mastigodiaptomus nesus* Bowman, 1986

–All urosomites with smooth dorsal surfaces (Fig. 4C); left Exp2 truncated or tapering distally, with apical denticles (plus hair-like setae) (Fig. 4G)9

9 Right Exp2 1.6 times longer than wide, with one enlarged hyaline membrane *Mastigodiaptomus siankaanensis* Mercado-Salas, Khodami, Kihara, Elías-Gutiérrez & Martínez-Arbizu, 2018

–Right Exp2 1.7–1.8 times longer than wide, with a narrow crescent-shaped sclerotisation (Figs. 3E and 4F)*Mastigodiaptomus alexei* sp. n.

10 Spine on antennular segment 8 enlarged, unarticulated, modified into a strong spiniform process (Fig. 13A); caudal surface of right Exp2 of P5 with two sclerotised projections plus one sclerotised longitudinal line (Figs. 11F and 13H)*Mastigodiaptomus cihuatlan* sp. n.

–Spine on antennular segment 8 extremely small, articulated (Figs. 4A, 8A and 15C); caudal surface of right Exp2 of P5 nude or with simple ornament11

11 Right Exp2 of P5 with naked surface; caudal surface of right basis of P5 with one triangular protuberance on distal margin and one rounded protuberance on medioproximal margin *M. cuneatus* Gutiérrez-Aguirre & Cervantes-Martínez, 2016

–Right Exp2 of P5 with one straight ridge, obliquely directed; caudal surface of right basis of P5 without protuberances, almost rectangular*M. amatitlanensis* Wilson, 1941

12 Left Exp2 of P5 distally truncated and denticulate; aculeus inserted subterminally into the right Exp2 of P5*M. texensis* Wilson, 1953

–Left Exp2 of P5 distally attenuated (triangular); aculeus inserted into the second third of the right Exp2 of P5 (Fig. 15G)13

13 Aculeus shorter than the length of right Exp2 of P5, with long spinules on medial margin; two short semi-circular lamellae on medial margin of right Exp2 of P5; second to forth urosomites with denticles on dorsal surfaces*M. suarezmoralesi* Gutiérrez-Aguirre & Cervantes-Martínez, 2013

–Aculeus subequal or longer than the length of the right Exp2 of P5, with short spinules on medial margin; one long, curved hyaline lamella on right Exp2 of P5 (Fig. 15G); dorsal surfaces of urosomites nude (Fig. 15F)14

14 Short spines on rostrum: 2.2–3.0 times longer than wide; cuticular surfaces of prosomites nude; 1.37–1.82 mm body length, including furcal rami*M. albuquerquensis* (Herrick, 1895)

–Long spines on rostrum: 3.5–5.0 times longer than wide (Fig. 16B); hair-like setae on ventral and distal margins from second to fifth prosomites (continuous or discontinuous rows) (Fig. 15B); 0.9–1.1 mm body length, including the furcal rami*M. patzcuarensis* (Kiefer, 1938)

### Females

1 Hair-like setae only on medial margin of furcal rami2

–Hair-like setae on both medial and lateral margins of furcal rami (Fig. 1C)3

2 Symmetric genital double somite, almost-parallel lateral margins, with short spines on left and right margins*Mastigodiaptomus purpureus*

–Asymmetric genital double somite, bulbose lateral margins, with short spines on left and right margins*M. reidae*

3 Almost symmetric genital double somite, almost-parallel lateral margins (Figs. 3A and 7A); two-segmented Enp of P54

–Asymmetric genital double somite, bulbose lateral margins (Figs. 11A and 14E); one or two-segmented Enp of P58

4 Left thoracic wing slightly shorter than right thoracic wing; A1 reaching proximal third of genital somite, left and right spines of the genital double somite placed at same level*Mastigodiaptomus maya*

–Left thoracic wing longer than right thoracic wing (Fig. 7A); A1 reaching beyond distal margin of genital double somite (Figs. 3A, 7A and 11A)5

5 A1 extending beyond caudal rami by last one or two segments; lateral margins of genital double somite parallel: right and left spines of the genital double somite placed at same level6

–A1 reaching anal somite or caudal rami; genital double somite asymmetric: right spine of genital double somite placed more proximal than left spine (Figs. 5C and 7F)7

6 Genital double somite 1.5 times longer than wide; antennules without hair-like setae on medial margin*Mastigodiaptomus texensis*

–Genital double somite 1.0–1.1 times longer than wide; antennules with hair-like setae on medial margin of segments 2 and 4–15*Mastigodiaptomus siankaanensis*

7 A1 reaching half length of anal somite (Fig. 7A); widest region of the body on first prosomite; length/width ratio of rostral spines = 2.0–2.1 (Fig. 7C); genital double somite 1.6–1.7 times longer than wide*Mastigodiaptomus ha* sp. n.

–A1 reaching distal margin of caudal rami (Fig. 3A); widest region of the body between second and third prosomites; length/width ratio of rostral spines = 2.5–2.6 (Fig. 5A); genital double somite 1.7–1.8 times longer than wide*Mastigodiaptomus alexei* sp. n.

8 One or two urosomites with a chitinous protuberance in the right posterolateral corner9

–Urosomites with straight posterior margins, no protuberances are present in the right posterolateral corner (Figs. 12E and 14E)10

9 Second urosomite with one acute protuberance in the right posterolateral corner; one-segmented Enp of P5*M. amatitlanensis*

–Genital double somite and second urosomite with chitinous protuberance in the right posterolateral corner; two-segmented Enp of P5*M. cuneatus*

10 Genital double somite, distal half, laterally swollen (Figs. 12E and 12F) or curved and wrinkled on the right lateral margin11

–Genital double somite, distal half, straight on the right lateral margin (Fig. 14E)13

11 Second and third urosomites fused ventrally, then the urosome is 2-segmented ventrally and 3-segmented dorsally (plus caudal rami) (Figs. 12F and 12G)*M. cihuatlan* sp. n.

–Urosomites unfused, then the urosome is 3-segmented ventral and dorsally (plus caudal rami) (Fig. 14E)12

12 Enp of P5, as long as medial margin of Exp1; distal half of genital double somite curved and wrinkled on the right lateral margin*M. suarezmoralesi*

–Enp of P5 shorter than the half length of the medial margin of Exp1; distal half of genital double somite swollen on the right lateral margin*M. montezumae*

13 Extremely small spines arranged in parallel rows on dorsal anal plate (Fig. 1C); left and right spines of genital double somite inserted at same level; ventral spine of right wing (of fifth prosomite) directed to the posterior region of the body*Mastigodiaptomus nesus*

–Dorsal anal plate nude; right spine of genital double somite inserted more proximally than left spine (Fig. 14E); ventral spine of right wing (of fifth prosomite) directed to the dorsal region of the body14

14 Short spines on rostrum: 1.5–3.6 times longer than wide; cuticular surface of prosomites smooth (or at most, extremely small hair-like setae on ventro-lateral surfaces); 1.4–1.8 mm of body length, including furcal rami*M. albuquerquensis*

–Long spines on rostrum: 3.6–4.0 times longer than wide (Figs. 14B and 16B); hair-like setae on ventral and distal margins from second to fifth prosomites (continuous or discontinuous rows) (Fig. 14A); 0.9–1.3 mm of body length, including furcal rami*M. patzcuarensis*

## Supplemental Information

10.7717/peerj.8416/supp-1 Supplemental Information 1 Representative haplotypes of all the genetic variants of COI within and between species.

10.7717/peerj.8416/supp-2 Supplemental Information 2 Id tree inferred, using the maximum likelihood method.

10.7717/peerj.8416/supp-3 Supplemental Information 3 Differences in morphology between eight *Mastigodiaptomus* species. Comparison of *Mastigodiaptomus* species described here and morphologically, genetically or distributionally similar species. For the new species the features were verified in the populations enlisted in material examined. (NE) refers to the number of spines or setae (number of all elements) on the mentioned segment. Undetermined data = ?

## Additional Information and Declarations

### DNA Deposition

The following information was supplied regarding the deposition of DNA sequences: DNA sequences are available at BOLD DOI 10.5883/DS-MMASTIGO.

### Data Availability

The following information was supplied regarding data availability: Specimen identification and accession number are available in Supplemental Files 1 and 2. DNA sequences are available at BOLD DOI: 10.5883/DS-MMASTIGO. Specimens are available in the Reference Collection at El Colegio de la Frontera Sur (ECOCH-CH-Z): 10312, 10313, 10314, 10315, 10316, 10317, 10318, 10319, 10320, 10321, 10322, 10323, 10324, 10325, 10328.

### New Species Registration

The following information was supplied regarding the registration of a newly described species: Publication LSID: urn:lsid:zoobank.org:pub:FEC66E33-E192-41C6-B3E1-C77C5D6705DE. Mastigodiaptomus alexei LSID: urn:lsid:zoobank.org:act:FFF6D019-9464-41E5-A992-E0C45B109CE7. Mastigodiaptomus ha LSID: urn:lsid:zoobank.org:act:0974ECE1-BF2A-4844-A5EA-4880CC957D1B. Mastigodiaptomus cihuatlan LSID: urn:lsid:zoobank.org:act:CF36F659-886A-4069-BF03-F1C300AE4FB8.
