# Supplementary material for: Remarks on Mastigodiaptomus (Calanoida: Diaptomidae) from Mexico using integrative taxonomy, with a key of identification and three new species
Source: PeerJ. 2020 Jan 29;8:e8416. doi: 10.7717/peerj.8416 (PMC6995272; doi:10.7717/peerj.8416)
Supplement: Supplemental Information 2 [file peerj-08-8416-s002.pdf]

BOLD TaxonID Tree

Title : Tree Result - Search: Seq Length(400 bp); Tax(Mastigodiaptomus); Include public records (221 records returned) (203 records selected)

Date : 22-Sep-2019

Data Type : Nucleotide

Distance Model : Kimura 2 Parameter

Marker : COI-5P

Colourization : [blue]=Stop Codons [red]=Contamination or misidentification

Label : Process ID

Label : Taxon

Label : Exact Site

Filter : length > 200bp only

Filter : exclude records flagged as misidentifications

Filter : exclude records with stop codons

Filter : exclude contaminants

Sequence Count : 203

Species count : 9

Genus count : 1

Family count : 1

Unidentified : 0

BIN Count : 19

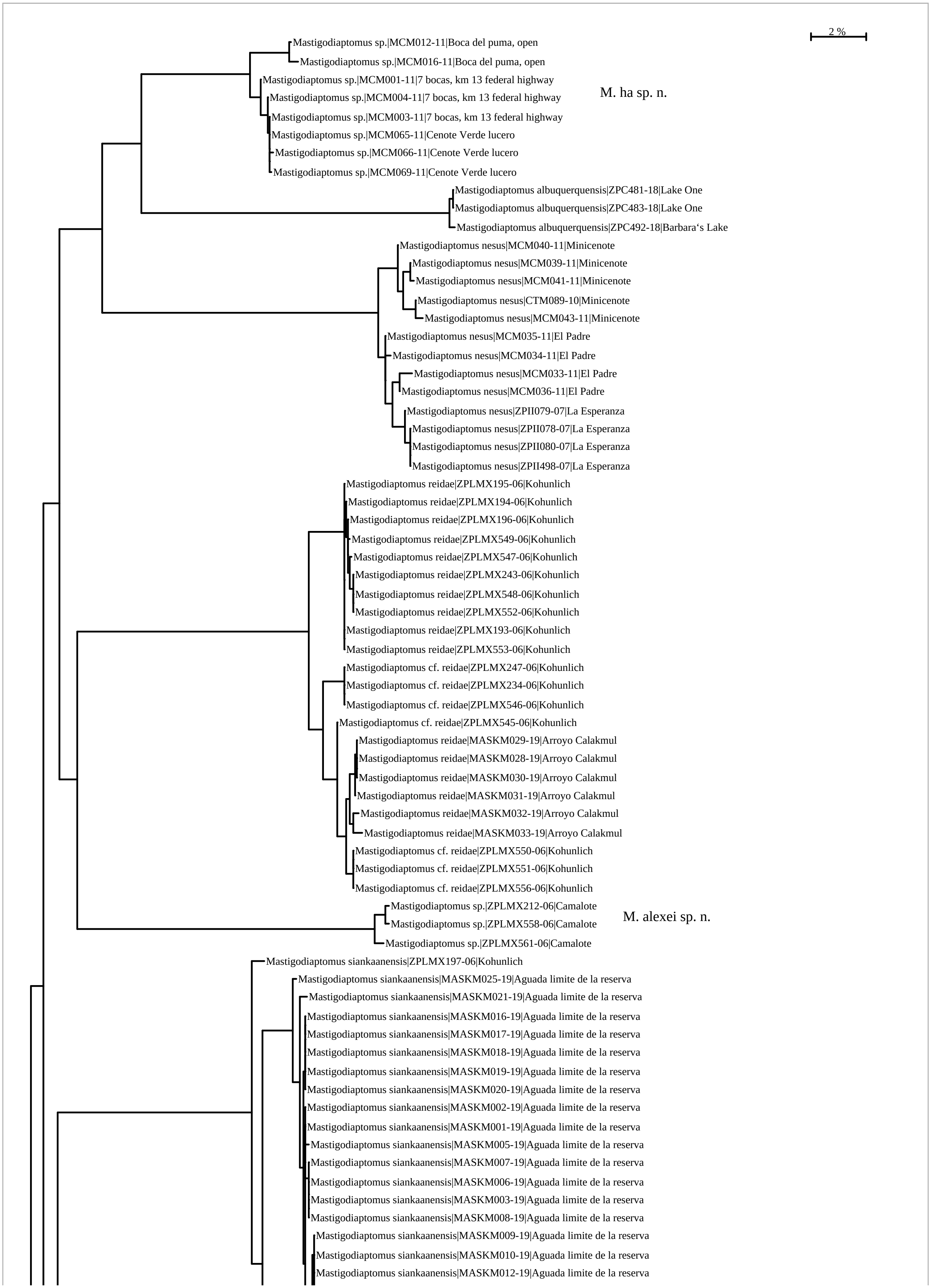
2 %
Mastigodiaptomus sp.|MCM012-11|Boca del puma, open
Mastigodiaptomus sp.|MCM016-11|Boca del puma, open
Mastigodiaptomus sp.|MCM001-11|7 bocas, km 13 federal highway
Mastigodiaptomus sp.|MCM004-11|7 bocas, km 13 federal highway
Mastigodiaptomus sp.|MCM003-11|7 bocas, km 13 federal highway
Mastigodiaptomus sp.|MCM065-11|Cenote Verde lucero
Mastigodiaptomus sp.|MCM066-11|Cenote Verde lucero
Mastigodiaptomus sp.|MCM069-11|Cenote Verde lucero
M. ha sp. n.
Mastigodiaptomus albuquerquensis|ZPC481-18|Lake One
Mastigodiaptomus albuquerquensis|ZPC483-18|Lake One
Mastigodiaptomus albuquerquensis|ZPC492-18|Barbara's Lake
Mastigodiaptomus nesus|MCM040-11|Minicenote
Mastigodiaptomus nesus|MCM039-11|Minicenote
Mastigodiaptomus nesus|MCM041-11|Minicenote
Mastigodiaptomus nesus|CTM089-10|Minicenote
Mastigodiaptomus nesus|MCM043-11|Minicenote
Mastigodiaptomus nesus|MCM035-11|El Padre
Mastigodiaptomus nesus|MCM034-11|El Padre
Mastigodiaptomus nesus|MCM033-11|El Padre
Mastigodiaptomus nesus|MCM036-11|El Padre
Mastigodiaptomus nesus|ZPII079-07|La Esperanza
Mastigodiaptomus nesus|ZPII078-07|La Esperanza
Mastigodiaptomus nesus|ZPII080-07|La Esperanza
Mastigodiaptomus nesus|ZPII498-07|La Esperanza
Mastigodiaptomus reidae|ZPLMX195-06|Kohunlich
Mastigodiaptomus reidae|ZPLMX194-06|Kohunlich
Mastigodiaptomus reidae|ZPLMX196-06|Kohunlich
Mastigodiaptomus reidae|ZPLMX549-06|Kohunlich
Mastigodiaptomus reidae|ZPLMX547-06|Kohunlich
Mastigodiaptomus reidae|ZPLMX243-06|Kohunlich
Mastigodiaptomus reidae|ZPLMX548-06|Kohunlich
Mastigodiaptomus reidae|ZPLMX552-06|Kohunlich
Mastigodiaptomus reidae|ZPLMX193-06|Kohunlich
Mastigodiaptomus reidae|ZPLMX553-06|Kohunlich
Mastigodiaptomus cf. reidae|ZPLMX247-06|Kohunlich
Mastigodiaptomus cf. reidae|ZPLMX234-06|Kohunlich
Mastigodiaptomus cf. reidae|ZPLMX546-06|Kohunlich
Mastigodiaptomus cf. reidae|ZPLMX545-06|Kohunlich
Mastigodiaptomus reidae|MASKM029-19|Arroyo Calakmul
Mastigodiaptomus reidae|MASKM028-19|Arroyo Calakmul
Mastigodiaptomus reidae|MASKM030-19|Arroyo Calakmul
Mastigodiaptomus reidae|MASKM031-19|Arroyo Calakmul
Mastigodiaptomus reidae|MASKM032-19|Arroyo Calakmul
Mastigodiaptomus reidae|MASKM033-19|Arroyo Calakmul
Mastigodiaptomus cf. reidae|ZPLMX550-06|Kohunlich
Mastigodiaptomus cf. reidae|ZPLMX551-06|Kohunlich
Mastigodiaptomus cf. reidae|ZPLMX556-06|Kohunlich
Mastigodiaptomus sp.|ZPLMX212-06|Camalote
Mastigodiaptomus sp.|ZPLMX558-06|Camalote
Mastigodiaptomus sp.|ZPLMX561-06|Camalote
M. alexei sp. n.
Mastigodiaptomus siankaanensis|ZPLMX197-06|Kohunlich
Mastigodiaptomus siankaanensis|MASKM025-19|Aguada limite de la reserva
Mastigodiaptomus siankaanensis|MASKM021-19|Aguada limite de la reserva
Mastigodiaptomus siankaanensis|MASKM016-19|Aguada limite de la reserva
Mastigodiaptomus siankaanensis|MASKM017-19|Aguada limite de la reserva
Mastigodiaptomus siankaanensis|MASKM018-19|Aguada limite de la reserva
Mastigodiaptomus siankaanensis|MASKM019-19|Aguada limite de la reserva
Mastigodiaptomus siankaanensis|MASKM020-19|Aguada limite de la reserva
Mastigodiaptomus siankaanensis|MASKM002-19|Aguada limite de la reserva
Mastigodiaptomus siankaanensis|MASKM001-19|Aguada limite de la reserva
Mastigodiaptomus siankaanensis|MASKM005-19|Aguada limite de la reserva
Mastigodiaptomus siankaanensis|MASKM007-19|Aguada limite de la reserva
Mastigodiaptomus siankaanensis|MASKM006-19|Aguada limite de la reserva
Mastigodiaptomus siankaanensis|MASKM003-19|Aguada limite de la reserva
Mastigodiaptomus siankaanensis|MASKM008-19|Aguada limite de la reserva
Mastigodiaptomus siankaanensis|MASKM009-19|Aguada limite de la reserva
Mastigodiaptomus siankaanensis|MASKM010-19|Aguada limite de la reserva
Mastigodiaptomus siankaanensis|MASKM012-19|Aguada limite de la reserva

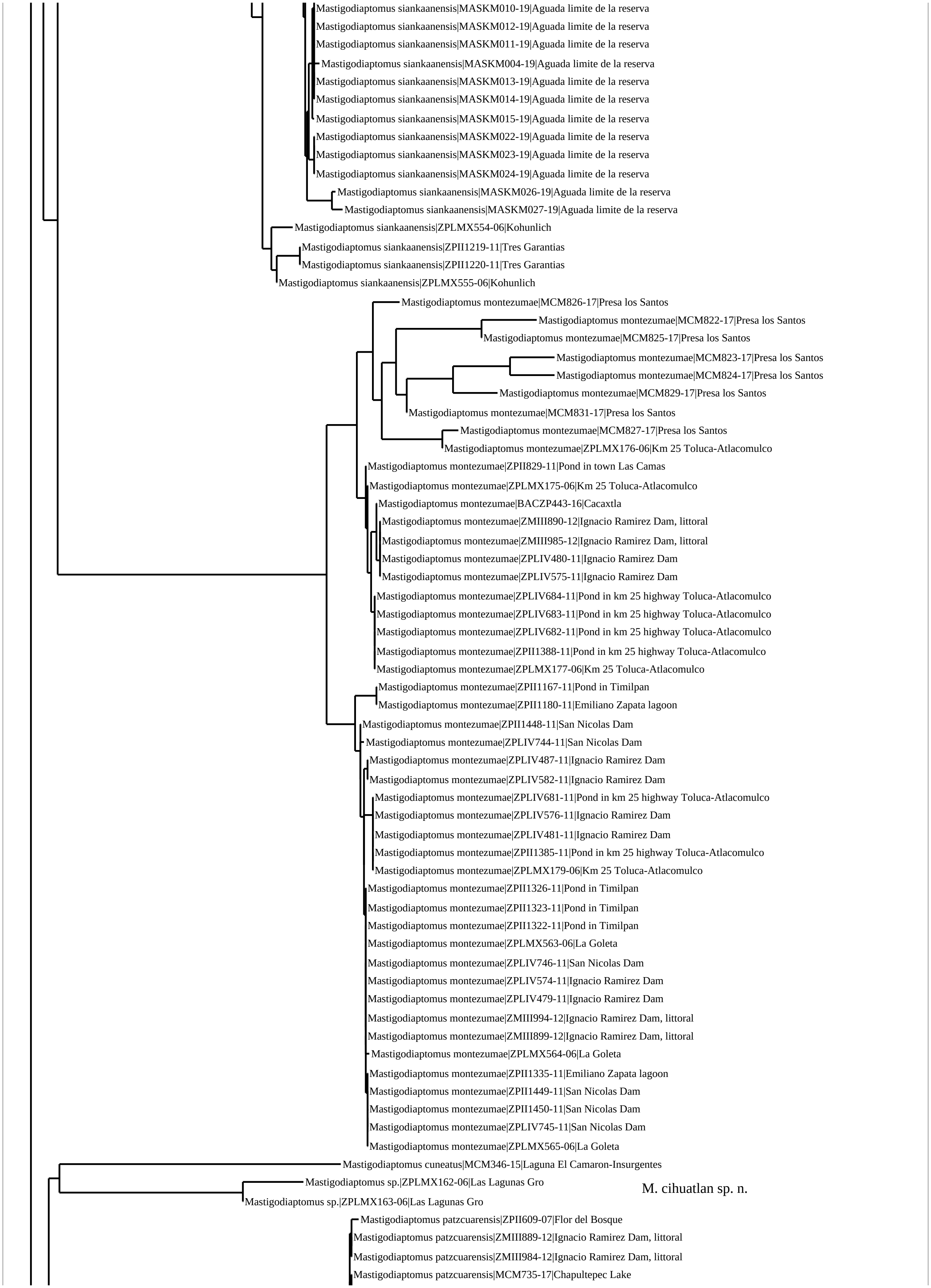
Mastigodiaptomus siankaanensis|MASKM010-19|Aguada limite de la reserva
Mastigodiaptomus siankaanensis|MASKM012-19|Aguada limite de la reserva
Mastigodiaptomus siankaanensis|MASKM011-19|Aguada limite de la reserva
Mastigodiaptomus siankaanensis|MASKM004-19|Aguada limite de la reserva
Mastigodiaptomus siankaanensis|MASKM013-19|Aguada limite de la reserva
Mastigodiaptomus siankaanensis|MASKM014-19|Aguada limite de la reserva
Mastigodiaptomus siankaanensis|MASKM015-19|Aguada limite de la reserva
Mastigodiaptomus siankaanensis|MASKM022-19|Aguada limite de la reserva
Mastigodiaptomus siankaanensis|MASKM023-19|Aguada limite de la reserva
Mastigodiaptomus siankaanensis|MASKM024-19|Aguada limite de la reserva
Mastigodiaptomus siankaanensis|MASKM026-19|Aguada limite de la reserva
Mastigodiaptomus siankaanensis|MASKM027-19|Aguada limite de la reserva
Mastigodiaptomus siankaanensis|ZPLMX554-06|Kohunlich
Mastigodiaptomus siankaanensis|ZPII1219-11|Tres Garantias
Mastigodiaptomus siankaanensis|ZPII1220-11|Tres Garantias
Mastigodiaptomus siankaanensis|ZPLMX555-06|Kohunlich
Mastigodiaptomus montezumae|MCM826-17|Presa los Santos
Mastigodiaptomus montezumae|MCM822-17|Presa los Santos
Mastigodiaptomus montezumae|MCM825-17|Presa los Santos
Mastigodiaptomus montezumae|MCM823-17|Presa los Santos
Mastigodiaptomus montezumae|MCM824-17|Presa los Santos
Mastigodiaptomus montezumae|MCM829-17|Presa los Santos
Mastigodiaptomus montezumae|MCM831-17|Presa los Santos
Mastigodiaptomus montezumae|MCM827-17|Presa los Santos
Mastigodiaptomus montezumae|ZPLMX176-06|Km 25 Toluca-Atlacomulco
Mastigodiaptomus montezumae|ZPII829-11|Pond in town Las Camas
Mastigodiaptomus montezumae|ZPLMX175-06|Km 25 Toluca-Atlacomulco
Mastigodiaptomus montezumae|BACZP443-16|Cacaxtla
Mastigodiaptomus montezumae|ZMIII890-12|Ignacio Ramirez Dam, littoral
Mastigodiaptomus montezumae|ZMIII985-12|Ignacio Ramirez Dam, littoral
Mastigodiaptomus montezumae|ZPLIV480-11|Ignacio Ramirez Dam
Mastigodiaptomus montezumae|ZPLIV575-11|Ignacio Ramirez Dam
Mastigodiaptomus montezumae|ZPLIV684-11|Pond in km 25 highway Toluca-Atlacomulco
Mastigodiaptomus montezumae|ZPLIV683-11|Pond in km 25 highway Toluca-Atlacomulco
Mastigodiaptomus montezumae|ZPLIV682-11|Pond in km 25 highway Toluca-Atlacomulco
Mastigodiaptomus montezumae|ZPII1388-11|Pond in km 25 highway Toluca-Atlacomulco
Mastigodiaptomus montezumae|ZPLMX177-06|Km 25 Toluca-Atlacomulco
Mastigodiaptomus montezumae|ZPII1167-11|Pond in Timilpan
Mastigodiaptomus montezumae|ZPII1180-11|Emiliano Zapata lagoon
Mastigodiaptomus montezumae|ZPII1448-11|San Nicolas Dam
Mastigodiaptomus montezumae|ZPLIV744-11|San Nicolas Dam
Mastigodiaptomus montezumae|ZPLIV487-11|Ignacio Ramirez Dam
Mastigodiaptomus montezumae|ZPLIV582-11|Ignacio Ramirez Dam
Mastigodiaptomus montezumae|ZPLIV681-11|Pond in km 25 highway Toluca-Atlacomulco
Mastigodiaptomus montezumae|ZPLIV576-11|Ignacio Ramirez Dam
Mastigodiaptomus montezumae|ZPLIV481-11|Ignacio Ramirez Dam
Mastigodiaptomus montezumae|ZPII1385-11|Pond in km 25 highway Toluca-Atlacomulco
Mastigodiaptomus montezumae|ZPLMX179-06|Km 25 Toluca-Atlacomulco
Mastigodiaptomus montezumae|ZPII1326-11|Pond in Timilpan
Mastigodiaptomus montezumae|ZPII1323-11|Pond in Timilpan
Mastigodiaptomus montezumae|ZPII1322-11|Pond in Timilpan
Mastigodiaptomus montezumae|ZPLMX563-06|La Goleta
Mastigodiaptomus montezumae|ZPLIV746-11|San Nicolas Dam
Mastigodiaptomus montezumae|ZPLIV574-11|Ignacio Ramirez Dam
Mastigodiaptomus montezumae|ZPLIV479-11|Ignacio Ramirez Dam
Mastigodiaptomus montezumae|ZMIII994-12|Ignacio Ramirez Dam, littoral
Mastigodiaptomus montezumae|ZMIII899-12|Ignacio Ramirez Dam, littoral
Mastigodiaptomus montezumae|ZPLMX564-06|La Goleta
Mastigodiaptomus montezumae|ZPII1335-11|Emiliano Zapata lagoon
Mastigodiaptomus montezumae|ZPII1449-11|San Nicolas Dam
Mastigodiaptomus montezumae|ZPII1450-11|San Nicolas Dam
Mastigodiaptomus montezumae|ZPLIV745-11|San Nicolas Dam
Mastigodiaptomus montezumae|ZPLMX565-06|La Goleta
Mastigodiaptomus cuneatus|MCM346-15|Laguna El Camaron-Insurgentes
Mastigodiaptomus sp.|ZPLMX162-06|Las Lagunas Gro
Mastigodiaptomus sp.|ZPLMX163-06|Las Lagunas Gro
M. cihuatlan sp. n.
Mastigodiaptomus patzcuarensis|ZPII609-07|Flor del Bosque
Mastigodiaptomus patzcuarensis|ZMIII889-12|Ignacio Ramirez Dam, littoral
Mastigodiaptomus patzcuarensis|ZMIII984-12|Ignacio Ramirez Dam, littoral
Mastigodiaptomus patzcuarensis|MCM735-17|Chapultepec Lake

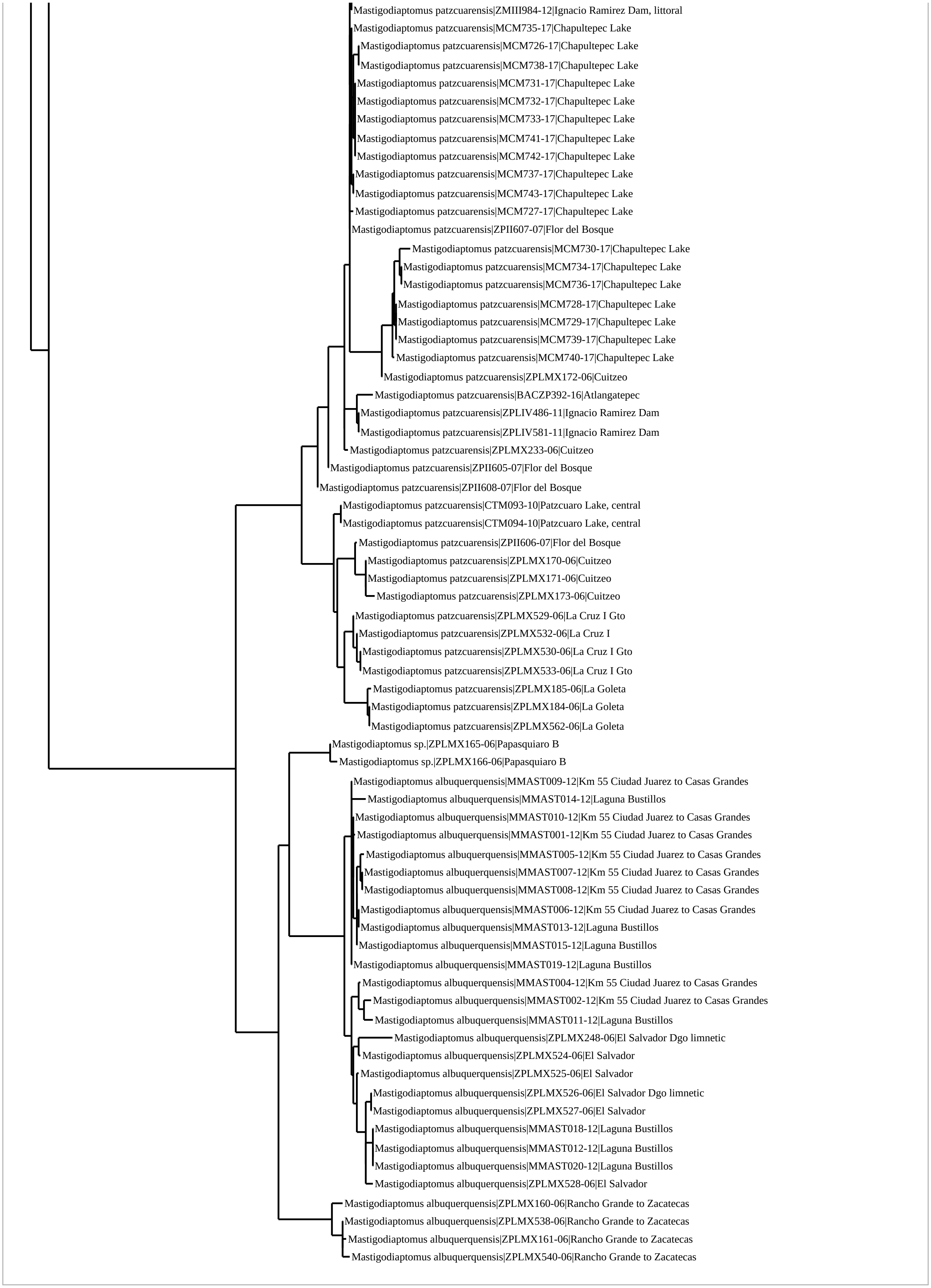
Mastigodiaptomus patzcuarensis|ZMIII984-12|Ignacio Ramirez Dam, littoral
Mastigodiaptomus patzcuarensis|MCM735-17|Chapultepec Lake
Mastigodiaptomus patzcuarensis|MCM726-17|Chapultepec Lake
Mastigodiaptomus patzcuarensis|MCM738-17|Chapultepec Lake
Mastigodiaptomus patzcuarensis|MCM731-17|Chapultepec Lake
Mastigodiaptomus patzcuarensis|MCM732-17|Chapultepec Lake
Mastigodiaptomus patzcuarensis|MCM733-17|Chapultepec Lake
Mastigodiaptomus patzcuarensis|MCM741-17|Chapultepec Lake
Mastigodiaptomus patzcuarensis|MCM742-17|Chapultepec Lake
Mastigodiaptomus patzcuarensis|MCM737-17|Chapultepec Lake
Mastigodiaptomus patzcuarensis|MCM743-17|Chapultepec Lake
Mastigodiaptomus patzcuarensis|MCM727-17|Chapultepec Lake
Mastigodiaptomus patzcuarensis|ZPII607-07|Flor del Bosque
Mastigodiaptomus patzcuarensis|MCM730-17|Chapultepec Lake
Mastigodiaptomus patzcuarensis|MCM734-17|Chapultepec Lake
Mastigodiaptomus patzcuarensis|MCM736-17|Chapultepec Lake
Mastigodiaptomus patzcuarensis|MCM728-17|Chapultepec Lake
Mastigodiaptomus patzcuarensis|MCM729-17|Chapultepec Lake
Mastigodiaptomus patzcuarensis|MCM739-17|Chapultepec Lake
Mastigodiaptomus patzcuarensis|MCM740-17|Chapultepec Lake
Mastigodiaptomus patzcuarensis|ZPLMX172-06|Cuitzeo
Mastigodiaptomus patzcuarensis|BACZP392-16|Atlangatepec
Mastigodiaptomus patzcuarensis|ZPLIV486-11|Ignacio Ramirez Dam
Mastigodiaptomus patzcuarensis|ZPLIV581-11|Ignacio Ramirez Dam
Mastigodiaptomus patzcuarensis|ZPLMX233-06|Cuitzeo
Mastigodiaptomus patzcuarensis|ZPII605-07|Flor del Bosque
Mastigodiaptomus patzcuarensis|ZPII608-07|Flor del Bosque
Mastigodiaptomus patzcuarensis|CTM093-10|Patzcuaro Lake, central
Mastigodiaptomus patzcuarensis|CTM094-10|Patzcuaro Lake, central
Mastigodiaptomus patzcuarensis|ZPII606-07|Flor del Bosque
Mastigodiaptomus patzcuarensis|ZPLMX170-06|Cuitzeo
Mastigodiaptomus patzcuarensis|ZPLMX171-06|Cuitzeo
Mastigodiaptomus patzcuarensis|ZPLMX173-06|Cuitzeo
Mastigodiaptomus patzcuarensis|ZPLMX529-06|La Cruz I Gto
Mastigodiaptomus patzcuarensis|ZPLMX532-06|La Cruz I
Mastigodiaptomus patzcuarensis|ZPLMX530-06|La Cruz I Gto
Mastigodiaptomus patzcuarensis|ZPLMX533-06|La Cruz I Gto
Mastigodiaptomus patzcuarensis|ZPLMX185-06|La Goleta
Mastigodiaptomus patzcuarensis|ZPLMX184-06|La Goleta
Mastigodiaptomus patzcuarensis|ZPLMX562-06|La Goleta
Mastigodiaptomus sp.|ZPLMX165-06|Papasquiaro B
Mastigodiaptomus sp.|ZPLMX166-06|Papasquiaro B
Mastigodiaptomus albuquerquensis|MMAST009-12|Km 55 Ciudad Juarez to Casas Grandes
Mastigodiaptomus albuquerquensis|MMAST014-12|Laguna Bustillos
Mastigodiaptomus albuquerquensis|MMAST010-12|Km 55 Ciudad Juarez to Casas Grandes
Mastigodiaptomus albuquerquensis|MMAST001-12|Km 55 Ciudad Juarez to Casas Grandes
Mastigodiaptomus albuquerquensis|MMAST005-12|Km 55 Ciudad Juarez to Casas Grandes
Mastigodiaptomus albuquerquensis|MMAST007-12|Km 55 Ciudad Juarez to Casas Grandes
Mastigodiaptomus albuquerquensis|MMAST008-12|Km 55 Ciudad Juarez to Casas Grandes
Mastigodiaptomus albuquerquensis|MMAST006-12|Km 55 Ciudad Juarez to Casas Grandes
Mastigodiaptomus albuquerquensis|MMAST013-12|Laguna Bustillos
Mastigodiaptomus albuquerquensis|MMAST015-12|Laguna Bustillos
Mastigodiaptomus albuquerquensis|MMAST019-12|Laguna Bustillos
Mastigodiaptomus albuquerquensis|MMAST004-12|Km 55 Ciudad Juarez to Casas Grandes
Mastigodiaptomus albuquerquensis|MMAST002-12|Km 55 Ciudad Juarez to Casas Grandes
Mastigodiaptomus albuquerquensis|MMAST011-12|Laguna Bustillos
Mastigodiaptomus albuquerquensis|ZPLMX248-06|El Salvador Dgo limnetic
Mastigodiaptomus albuquerquensis|ZPLMX524-06|El Salvador
Mastigodiaptomus albuquerquensis|ZPLMX525-06|El Salvador
Mastigodiaptomus albuquerquensis|ZPLMX526-06|El Salvador Dgo limnetic
Mastigodiaptomus albuquerquensis|ZPLMX527-06|El Salvador
Mastigodiaptomus albuquerquensis|MMAST018-12|Laguna Bustillos
Mastigodiaptomus albuquerquensis|MMAST012-12|Laguna Bustillos
Mastigodiaptomus albuquerquensis|MMAST020-12|Laguna Bustillos
Mastigodiaptomus albuquerquensis|ZPLMX528-06|El Salvador
Mastigodiaptomus albuquerquensis|ZPLMX160-06|Rancho Grande to Zacatecas
Mastigodiaptomus albuquerquensis|ZPLMX538-06|Rancho Grande to Zacatecas
Mastigodiaptomus albuquerquensis|ZPLMX161-06|Rancho Grande to Zacatecas
Mastigodiaptomus albuquerquensis|ZPLMX540-06|Rancho Grande to Zacatecas
