# Supplementary material for: Remarks on Mastigodiaptomus (Calanoida: Diaptomidae) from Mexico using integrative taxonomy, with a key of identification and three new species
Source: PeerJ. 2020 Jan 29;8:e8416. doi: 10.7717/peerj.8416 (PMC6995272; doi:10.7717/peerj.8416)
Supplement: Supplemental Information 3 — Comparison of Mastigodiaptomus species described here and morphologically, genetically or distributionally similar species. For the new species the features were verified in the populations enlisted in material examined. (NE) refers to the number of spines or setae (number of all elements) on the mentioned segment. Undetermined data = ? [file peerj-08-8416-s003.docx]

| Character | *M. nesus* (paratypes) (Bowman, 1986) | *M. texensis* s. str. (Wilson, 1953) | *M. alexei* sp. n. | *M. ha* sp. n. | *M. cihuatlan* sp. n. | *M. maya* (Suárez-Morales and Elías-Gutiérrez 2000) | *M. reidae* (Suárez-Morales and Elías-Gutiérrez 2000) | *M. siankaanensis* (Mercado-Salas et al., 2018) |
| --- | --- | --- | --- | --- | --- | --- | --- | --- |
| ♀, ♂; Antennae, Enp2, (NE) on medial lobe + terminal lobe | 8 + 7 | ? | 9 + 7 | 9 + 7 | 7 + 7 | 6 + 6 | 8 + 7 | 9 + 7 |
| ♀, ♂; Maxillule, praecoxal arthrite, (NE) on anterior group + posterior group | 8 + 4 | ? | 9 + 4 | 11 + 4 | 10 + 4 | 9 + 4 | 10 + 4 | 11 + 4 |
| ♀, ♂; Maxilla, (NE) on praecoxal + coxal lobes | 5, 3 + 3, 3 | ? | 4, 3 + 3, 3 | 4, 3 + 3, 3 | 5, 3 + 3, 3 | 4, 3 + 2, 3 | ? | 5, 3 + 2, 3 |
| ♀, ♂; Maxilla, (NE) on endopodites | 1, 2, 3 | ? | 1, 1, 3 | 1, 2, 3 | 1, 2, 3 | 1, 1, 3 | ? | 1, 4 |
| ♀, Antennules | Beyond caudal rami by last four segments | Beyond caudal rami by last two segments | Reaches distal margin of caudal rami | Reaches medial length of anal somite | Beyond caudal rami by last six segments | Reaches proximal third of genital somite | Reaches distal margin of genital somite | Beyond caudal rami by last one or two segments |
| ♀, Setules on Fu margin | On medial and lateral margins | On medial and lateral margins | On medial and lateral margins | On medial and lateral margins | On medial and lateral margins | On medial and lateral margins | On medial margin | On medial and lateral margins |
| L/W, Rostral spines | 2.5 (♀); 2.2 (♂) | ND | 2.6 (♀); 2.3 (♂) | 2.1 (♀); 2.0 (♂) | 4.1 (♀); 4.0 (♂) | 2.0 (♀); 1.4 (♂) | 3.0 (♀); 2.33 (♂) | ND |
| ♀, Lateral margins of genital somite | Left margin slightly pronounced; right margin pronounced | Parallel | Almost parallel | Almost parallel | Left margin slightly pronounced; right margin pronounced | Almost parallel: right margin longer than left margin | Left margin slightly pronounced; right margin almost straight | Parallel |
| ♀, Lateral spines on genital double somite | Almost symmetric: same level | Almost symmetric: same level | Asymmetric: right spine more proximal than left spine | Asymmetric: right spine more proximal than left spine | Asymmetric, twisted: right spine more proximal than left spine. Left spine towards dorsal surface. Right margin produced | Almost symmetric: same level | Slightly asymmetric: right spine more proximal than left spine | Almost symmetric: same level |
| ♀, L/W, Genital double somite | 1.2-1.5 | 1.5 | 1.7-1.85 | 1.6-1.7 | 1.2-1.6 | 1.75 | 2.0 | 1.08-1.1 |
| ♀, P5 L Exp1/Enp | 1.6-1.9 | 1.0 | 1.1-1.2 | 1.1-1.2 | 0.8-0.9 | 1.3 | 1.6 | 1.5 |
| ♂, Right A1, spinal process on segment 10 | Short: reaches distal margin of the bearing segment | Extremely small: does not reach distal margin of the bearing segment | Short: reaches distal margin of the bearing segment | Short: reaches distal margin of the bearing segment | Long: beyond distal margin of the bearing segment | Short: reaches distal margin of the bearing segment | Short: reaches distal margin of the bearing segment | Spinal process absent.  The strong spine does not reach distal margin of the bearing segment |
| ♂, Right A1, spinal process on segment 11 | Almost as long as or as long as the segment 11 width | Almost as long as the segment 11 width | 1.25-1.3 times longer than the segment 11 width | 1.50-1.55 times longer than the segment 11 width | 1.9-2.1 times longer than the segment 11 width | 1.2 times longer than the segment 11 width | 1.6 times longer than the segment 11 width | Almost as long as the segment 11 width |
| ♂, Right A1, spinal process on segment 16 | Reduced, on half of the segment | Reduced, on distal half | Reduced, on proximal half | Reduced, on proximal half | Reduced, on half of the segment | Reduced, on half of the segment | Strongly developed, on half of the segment | Reduced, on half of the segment |
| ♂, Right A1, spinal process on segment 20 | Fang-like, curved, reaches half-length of segment 21 | Fang-like, curved, reaches half-length of segment 21 | Fang-like, curved, reaches the distal third of segment 21 | Fang-like, curved, reaches half-length of segment 21 | Fang-like, angled, reaches half-length of segment 21 | Knob-like, reaches proximal third of segment 21 | Fang-like, curved, reaches half-length of segment 21 | Fang-like, curved, reaches the distal margin of segment 21 |
| ♂, Left P5, L Exp1/Exp2 | 1.57-2.0 | 3.33 | 2.4-2.7 | 2.5-2.6 | 1.5-1.6 | 2.33-2.5 | 2.0 | 1.7 |
| ♂, P5, right basis armament | One semi-circular lamella on medial margin | One protusion with a crescent-shaped sclerotized lamella + one angled lamella, distally directed on medial margin | One high protrusion + one rounded hyaline membrane on medial margin | One low protrusion + one rounded hyaline membrane on medial margin | One angled basal process + one long hyaline membrane that extends along medial margin of the segment | One basal rounded protrusion | One basal subrectangular protrusion, one basal, bulb-like process, and one semi-circular process on medial margin | One low protrusion |
| ♂, P5, right Exp2 armament (posterior surface) | One long, quadrangular sclerotization | One crescent-shaped sclerotization | One crescent-shaped sclerotization | None, Exp2 smooth | Two rectangular basal sclerotizations + one sclerotized ridge triangular-shape distally | One low, rounded protuberance proximally | One chitinous semi-circular process on proximal third | One slightly curved hyaline membrane |
| ♂, P5, right Exp2, L distal spine/ L Exp2  Segment | 2.2- 2.44 | 2.62 | 2.9-3.3 | 2.8-3.2 | 2.3-2.4 | 1.9-2.1 | 2.3 | 2.1-2.2 |
| ♂, P5, right Enp | Not reaching distal margin of right Exp1 | Not reaching distal margin of right Exp1 | Beyond distal margin of right Exp1 | Beyond distal margin of right Exp1 | Beyond distal margin of right Exp1 | Reaches distal margin of Exp1 | Beyond distal margin of right Exp1 | Beyond distal margin of right Exp1 |
| ♂, Right projection, preanal segment (dorsal) | Rounded | Rounded | With angled protuberance | Rounded | Quadrangular | Quadrangular | With angled protuberance | With angled protuberance |
